# Descriptive and multivariate analysis of the pig sector in Georgia and its implications for disease transmission

**DOI:** 10.1371/journal.pone.0202800

**Published:** 2018-08-24

**Authors:** Daniel Beltrán-Alcrudo, Esther A. Kukielka, Nienke de Groot, Klaas Dietze, Mikheil Sokhadze, Beatriz Martínez-López

**Affiliations:** 1 Regional Office for Europe and Central Asia, Food and Agriculture Organization, Budapest, Hungary; 2 Center for Animal Disease Modeling and Surveillance (CADMS), Department of Medicine & Epidemiology, School of Veterinary Medicine, University of California, Davis, CA, United States of America; 3 Utrecht University, Utrecht, The Netherlands; 4 Institut für Epidemiologie, Friedrich-Loeffler-Institut (FLI), Greifswald—Insel Riems, Germany; 5 National Food Agency, Tbilisi, Georgia; 6 FAO Representation in Georgia, Food and Agriculture Organization, Tbilisi, Georgia; Institute of Subtropical Agriculture, Chinese Academy of Sciences, CHINA

## Abstract

**Background:**

Georgia is a country in the Caucasus region with a traditional backyard and highly variable pig farming system. The practices of such sectors have seldom been described and analyzed to better understand their implication in the introduction and spread of infectious pig diseases. Moreover, the Georgian pig sector was badly hit by an epidemic of African swine fever in 2007 that quickly spread throughout the region.

**Materials and methods:**

We interviewed 487 pig farmers and 116 butchers using closed questionnaires on socioeconomic issues related to pig production, husbandry practices, biosecurity, marketing and movements, and disease awareness. Surveys were conducted in four regions of Georgia and descriptive statistics were computed. Factorial analyses of mixed data and hierarchical clustering on principal components were applied to study the relationship among collected variables for both farmers and butchers.

**Results:**

Results show that pig farming in Georgia is a non-professional sector, highly heterogeneous by region, characterized by smallholdings of few animals, with low inputs, outdated technologies, and poor biosecurity, which all translates into low outputs and productivity. The hierarchical clustering on principal components confirmed that there are five major production and husbandry strategies, which match the four regions where the study was conducted.

**Conclusions:**

Our results are the first step to quantify biosecurity gaps and risky behaviours, develop risk profiles, and identify critical control points across the market chain where to implement mitigation measures. This study provides the baseline information needed to design realistic and sustainable prevention, surveillance and control strategies.

## Introduction

**Fig 1:**
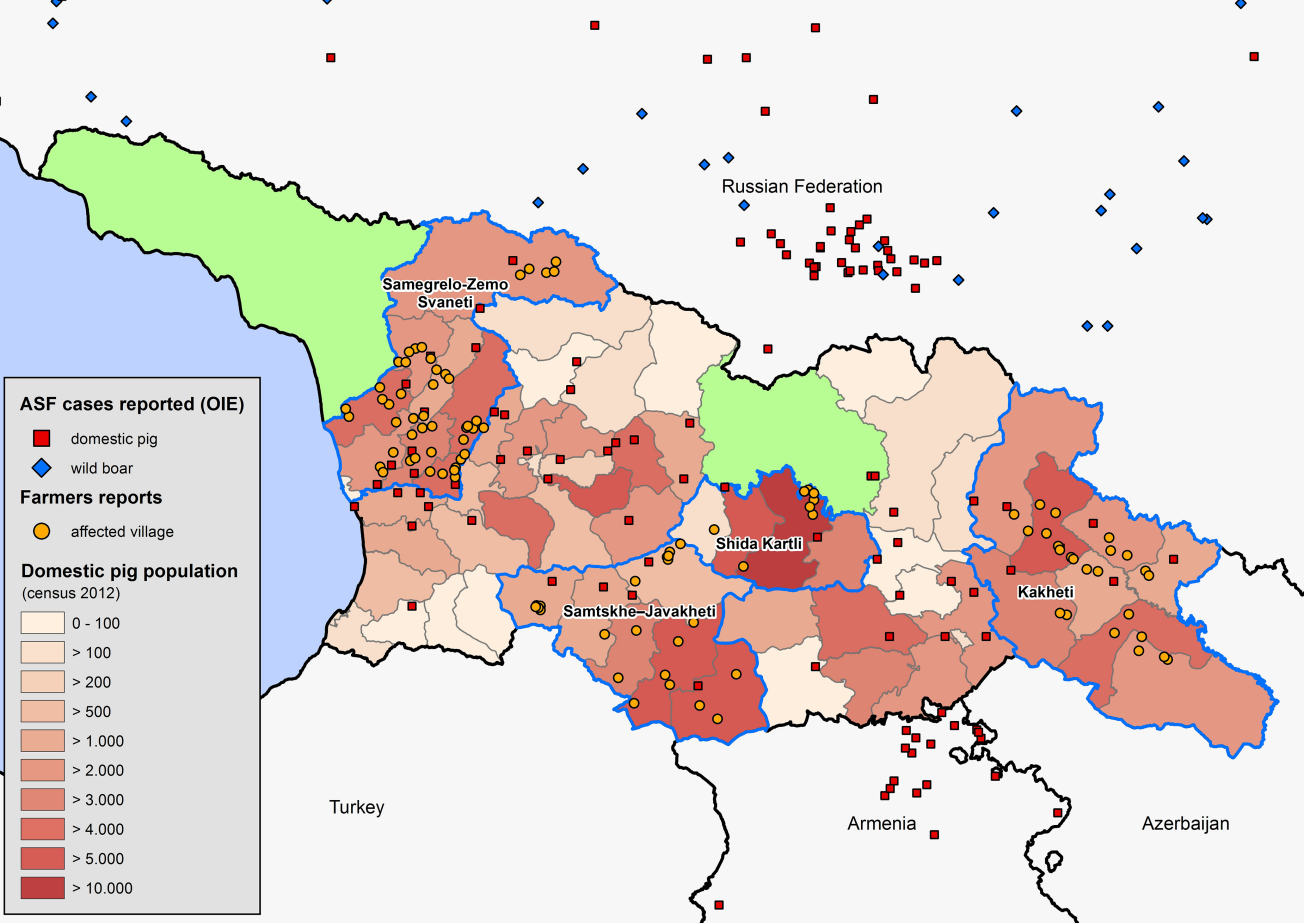
Map of Georgia showing study areas, African swine fever outbreaks and swine density. Study areas are delimited by a blue line; Politically disputed regions are depicted in light green.

Georgia is a country in the Caucasus region of Eurasia. The estimated number of pigs in Georgia has fluctuated considerably over the years, i.e. with over one million heads in the 1980s, numbers gradually declined to 343,500 in 2006, to dramatically drop to 86,400 in 2008, the year after the epidemic of African swine fever (ASF). Since then, the sector has doubled until the 204,300 heads recorded in 2012, when this study was conducted [1]. This translates into 11,800 tons of pork, which is the most expensive meat, i.e. 12.24 GEL/Kg together with beef (12.10 GEL/Kg; around 5 USD/Kg). The import of pork in 2012 amounted for 6,883 tons [2]. Most pigs are managed under a backyard production system (non-professional pig holdings) with few animals, low biosecurity (often scavenging systems) and outdated technologies [3]. Rearing pigs is a very common traditional practice in rural areas, representing an important meat source and often generating valuable cash income for farmers. Pig farming is spread heterogeneously throughout the country, with the highest densities found in the eastern and the western plains of the country and the lowest densities located in the mountainous areas along the border with the Russian Federation (north) and along the borders with Turkey and Armenia (south) (Fig 1). Despite the relative small size of Georgia (69,700 km^2^), husbandry systems vary greatly across the country in terms of the production system and the preferred final products, e.g. cured hams, piglets, etc. Due to the small-scale production, slaughtering and butchering generally takes place on farm, i.e. home-slaughtering; thus, lacking veterinary meat inspection before selling (which is only ensured for meat destined to urban supermarkets).

On 5 June 2007, Georgia officially notified its first occurrence of ASF in pigs to the World Organisation for Animal Health (OIE) [4]. ASF is a haemorrhagic fever characterized by high lethality, which spreads mostly through direct contact (also involving wild boar) and the ingestion of contaminated products, e.g. pork, and against which there is no effective vaccine or treatment available. Except for endemic Sardinia, this was the first ever occurrence of ASF outside of Africa since the last isolated outbreak in Portugal in 1999 [5]. All evidence so far indicates that the virus was probably introduced into the Black Sea port of Poti by improperly disposed catering waste from international ships carrying contaminated pork or pork products [3]. Scavenging pigs probably got infected by accessing the dumped port waste. Afterwards, the disease spread eastwards and north following the main transportation routes and, by the second week of June of the same year, 52 out of 65 districts were affected [3]. Most pigs affected were on open grazed fields or in free range systems. Soon, the infection spread to neighbouring countries, with multiple outbreaks in Armenia in August, wild boar affected in the Chechnya Republic in the Russian Federation, and a single outbreak in a village in north-west Azerbaijan in January 2008 [6–8]. Spreading and persistence in the region was mainly associated with smallholder/backyard production and the movement of infected pork products.

Disease prevention and control is most challenging in backyard settings due to the lower levels of awareness among rural communities, low biosecurity, poor compliance to livestock related regulations (reporting, movement control, certifications, vaccination, etc.) and lack of animal identification and traceability. Little is known about these systems and trade networks due to their mostly informal nature. Such information, however, is crucial to develop targeted, realistic and sustainable disease prevention and control interventions.

This study aims to gain knowledge (through field surveys in the form of questionnaires) about the Georgian pig production system by understanding people’s behaviours, trade patterns and management practices involved in smallholder pig production and processing, which are largely unknown and highly diverse within the country. The analysis of questionnaires will allow to identify and quantify biosecurity gaps and risky behaviours, develop risk profiles, and identify critical control points across the market chain where mitigation measures could be implemented.

## Materials and methods

Two questionnaires were developed, one for pig farmers and other for butchers, to gather information about biosecurity and husbandry practices, market chains, wild boar interactions, awareness status and socio-economic aspects.

### Study site

Georgia is a country in the Caucasus region of Eurasia, at the crossroads of Western Asia and Eastern Europe, with boundaries to the Black Sea (west), the Russian Federation (north), Turkey and Armenia (south), and Azerbaijan (southeast). Partly due to its very mountainous nature, there are 12 different climatic zones. Together with the 49 soil types, this results in a wide variety of ecological and climatic zones [9]. Georgia’s population in 2016 was 4.498 million in 2012 (density of 64.5/km^2^) (GEOSTAT, 2017), of which 42.8 percent is rural (National Statistics Office of Georgia, 2013). Georgia is a highly agricultural country, with 43.4% of the territory designated as agricultural land and 52% of the country’s labor force dedicated to agriculture [9]. Animal husbandry accounts to 49% of the agricultural sector [1]. The study was conducted in four regions of Georgia: Samtskhe-Javakheti (SJ), Kakheti, Samegrelo-Zemo Svaneti (SZS) and Shida Kartli (SK).

### Ethical statement

FAO followed the principles of the declaration of Helsinki and the Belmont report when designing and implementing the survey. The Institutional Review Board (IRB) of UC Davis Administration issued an exemption from the requirement for IRB review, the reasons being that the surveys would not elicit responses that would place the respondents at risk if obtained by individuals not associated with the research. The exemption criteria are available at 45 CFR 46.101(b)(2)–U.S. Code of Federal Regulation, Protection of human subjects. All the interviewed farmers and butchers were informed of the study purpose, and of the facts that participation in the interviews was voluntary and they could drop from the study at any time.

### Background data gathering

To gather information about the pig sector and value chains, one-day workshops were organized in three regions known to have markedly different production systems: Kakheti in the east (24 participants); Guria in the west (5 participants), and Racha Lechkhumi in the north (13 participants). Participants included a mix of representatives from different stakeholder groups: backyard pig owners, commercial farmers, private and state veterinarians, meat vendors, butchers and hunters. They voluntarily enrolled and were selected by the state veterinarian in each region. Flow charts were drawn-up identifying the main actors, locations, commodities, and seasonality of the pig and pork production chain. The geographical location of commercial farms, live animal markets, free-ranging areas and wild boar habitats were mapped. The ASF epidemic was also discussed, including the prevention and control measures in place, the lessons learned and the effects in the market chain. This information was used to design the questionnaires.

The following background information was collected from the villages in the four selected regions: 1) whether in a mountainous area; 2) demographic data (i.e. number of inhabitants, pig owners, pigs and butchers); and 3) the most prevalent farm size/s, i.e. small (<5 pigs), medium (5–10) or large farmer (>10).

### Questionnaire design

Semi-structured questionnaires (one for pig farmers and one for butchers) were originally written in English and subsequently translated into Georgian and Russian (to also target ethnic minorities in SJ). The questionnaires included five sections: (1) socioeconomic issues related to pig production, (2) husbandry practices, (3) biosecurity, (4) marketing and movements, and (5) disease awareness, plus the names of the interviewee and interviewer, the farm location (village/municipality/region), and the date of the interview. All questions referred to the period over the previous 12 months.

The questionnaires were pilot-tested in Kakheti, where 55 questionnaires (30 farmers and 25 butchers) were collected from all eight municipalities by state veterinarians while conducting serosurveillance activities. The questionnaires were amended according to the feedback and gaps (S1 Appendix). Results from the pilot were not included in the final analysis.

### Sample selection

A cross-sectional study was conducted in September-November 2012 in 168 villages of 25 municipalities within the four regions where veterinary associations (hired to deliver the questionnaires) are present. A total of 603 questionnaires were conducted, distributed among regions and the interviewees’ profession as per Table 1.

**Table 1:** Summary of questionnaires’ implementation in pig farmers and butchers in four regions of Georgia, in 2012.

Region	Municipalities selected	Villages	Interviewers’ trainings	End of interviews	Vets hired	Interviewed farmers/ butchers/total
**Kakheti**	Sagarejo, Gurjaani, Telavi, Signagi, Akhmeta, Dedoflistskaro, Kvareli and Lagodekhi	42	20.09.2012	27.10.2012	8	120 / 30 / 150
**Samegrelo-Zemo Svaneti**	Martvili, Zugdidi, Senaki, Khobi, Abasha, Chkhorotsku, Tsalenjikha and Mestia	47	28.09.2012	30.10.2012	9	122 / 31 / 153
**Samtskhe-Javakheti**	Aspindza, Adigeni, Ninotsminda, Akhalkalaki, Akhaltsikhe and Borjomi	37	26.09.2012	06.11.2012	6	125 / 25 / 150
**Shida-Kartli**	Gori, Kareli, Khashuri and Kaspi	43	21.09.2012	06.10.2012	4	120 / 30 / 150
**Total**	25	168			27	487 / 116 / 603

Villages selection followed the following criteria: 1) the biggest town in the municipality (believed to play a more important role in the regional pig and pork value chains); 2) both mountain and non-mountain villages; and 3) evenly distributed among municipalities. The number of interviews was determined by the funds available.

### Data collection

Questionnaires were conducted by private veterinarians belonging to veterinary associations, who were selected based on their access to butchers and the villages they serviced. Veterinarians reported to be less likely to deliver or less trustworthy were excluded. Prior questionnaire implementation, training sessions were organized in each region for the interviewers, covering the survey goal, content, schedule and basic interview techniques.

The interviewers were instructed to conduct approximately five questionnaires per village according to the following criteria: 1) 20% of the questionnaires with butchers and 80% with farmers; 2) select those farmers with more pigs (>5 animals), who are likely to play a more active role in the value chain; 3) select only butchers who work with Georgian pork. If butchers also owned pigs, they also were requested to answer to the farmer questionnaire (and *vice versa*). Face-to-face interviews took 20–30 minutes for 41 questions (with several subsections) for the farmer’s questionnaire and 10–20 minutes for 24 questions for the butcher’s questionnaire.

An Excel database was developed and questionnaires were scanned. Prior to data analysis, some farmers had their answers checked by phone due to missing values and inconsistencies.

### Data analysis

Descriptive statistics were computed from the questionnaire results (S1 and S2 Databases). Two factorial analyses of mixed data (FAMD) were carried out to study the relationship amongst collected variables and individuals (i.e. farms and butchers) in a descriptive graphical manner. FAMD is a principal component method (non-linear multivariate approach) that allows for analysis of both quantitative and qualitative data. This methodology is recommended when there is an interest on leaving quantitative variables as such (without having to transform them into qualitative and therefore without the need of splitting their variation interval into classes, thus loosing information in the process) and when the number of individuals in the study is larger than 100 (in our case n = 487 for farmers, n = 116 for butchers) [10].

In the case of the farmers’ questionnaire, out of 75 available variables, similar variables were combined and variables lacking variability of response were deleted.

FAMD was also used as a pre-processing step for two hierarchical clustering on principal components (HCPC): one for farmers, one for butchers. HCPC is an exploratory data analysis method that allows to cluster observed individuals (i.e. farmers, butchers) according to their similarities in collected variables. When HCPC is computed, a hierarchical tree is constructed using the multidimensional Euclidean distance and the Ward's agglomeration method [10]. In our case, the number of clusters chosen was selected based on the bar plot of the gain in within-inertia as suggested by the HCPC analysis. This analysis suggests a final cluster division of "Q clusters when the between-inertia among Q-1 and Q clusters is much larger than the one between Q and Q+1 clusters" [10]. Statistical significance was assumed at a p-value<0.05. The most important variables taken into account towards the decision of the cluster formation are presented according to their v.test value. A v.test = |1.96| is equivalent to a p-value<0.05. The sign of the v.test indicates whether the average of that variable in the cluster is larger (positive sign) or smaller (negative sign) than the average of that variable in the complete dataset. FactoMineR package in R language was used for both the FAMD and the HCPC analysis [11].

## Results

Thirty out of 41 questions and 22 over 24 questions had response rates over 95% for farmers and butchers, respectively.

### Farmers

Detailed results are presented in S1 Table.

#### Socio economic aspects

Farmers kept an average of 11.0 of pigs of all types (2.8 pigs kept for fattening and 5.8 piglets). Most farmers do not own a boar (52.4%) and keep 1 to 3 reproductive sows (64.5%). Still, 25.9% of them kept no sows, meaning that they just buy piglets or half fattened pigs for fattening and home-slaughtering. Pig farming is the major economic activity (defined as >50% of household income) only for 9.3% of farmers. The average household income was 23.4% (±23.7), implying that most farmers have other jobs or major sources of income. The very few farmers who also work as butchers (3.7%) were requested to also answer the butcher’s questionnaire. Taking care of the pigs is a task often shared by more than one person (66.3%), usually family members, i.e. the wife (69.6%) and husband (75.8%), but in SJ and SK, kids also play an important role. Only 1.9% of pig farmers hired personnel, mostly in Kakheti (4.2%).

#### Production system

**Fig 2:**
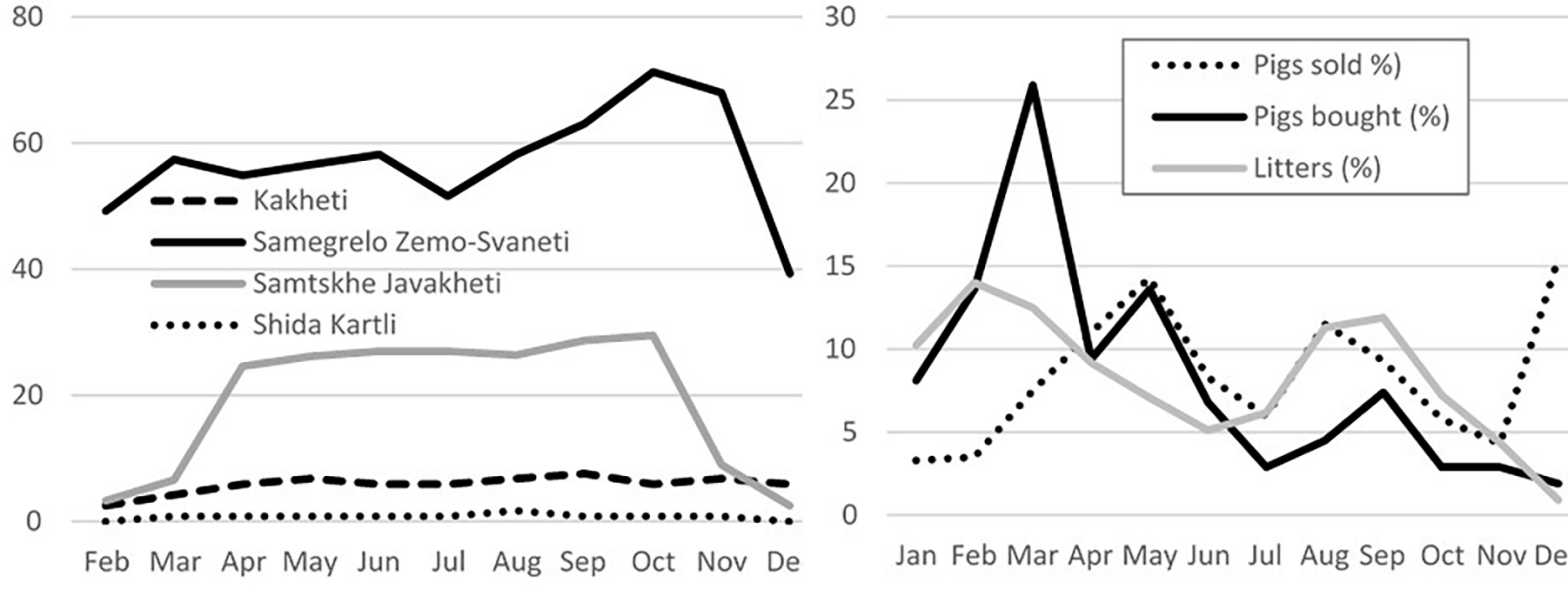
Pig production seasonality graphs in four regions Georgia: A) Proportion of pig scavenging by region per month by region (%); B) Proportion of pig transactions per month (%).

Local breeds are very popular (86.2% of animals) and only 10.1% of farmers keep exclusively commercial breeds. Although most interviewed farmers (67.6%) keep their pigs enclosed all year round, confinement of pigs is highly variable across the country, with scavenging systems ranging from 1.7% to 87.7% (SZS) of farms depending on the region. In SJ, scavenging is allowed from April to October by 28.8% of farmers (Fig 2A). Littering follows a marked seasonality with two peaks in February-March and August-September, and its lowest point in December (Fig 2B). The February peak in SK represents 25.3% of the total litters. The number of piglets per sow are highly variable (range = 0.75–27.0), for an average of 7.9 offspring per sow per year. Villages in SJ are located the highest (over 1,600 m over sea level).

#### Health management

Most farms experienced no mortality at all (68.7%). Pigs and piglets were rarely reported to disappear (2.9% of farms), with only SZS, where most free-ranging of pigs occur, reporting significant figures (9% of farms reported at least one missing animal). Vaccination against classical swine fever was the most common, followed by erysipela, pasteurellosis and Aujeszky’s disease. Over 80% of farmers reported consulting a veterinarian at least once over the past 12 months (8.1±10.7 consultations per year). In the event of encountering a sick pig, most farmers reported to treat the animal themselves (76.3%) and/or consulted a veterinarian (80.4%). When encountering deaths, most farmers bury the animals (66.3%) or dispose them in a pit (28.3%). However, this question had a low response rate (34.0%).

**Fig 3:**
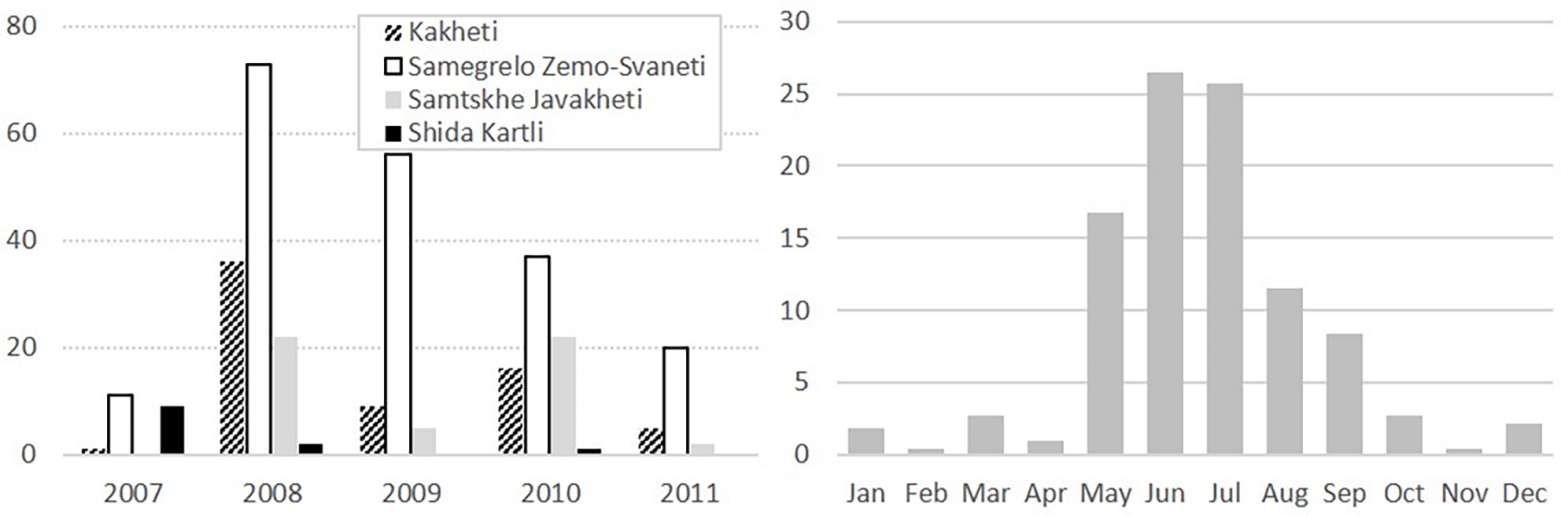
**Distribution of reported African swine fever compatible outbreaks in four regions in Georgia by year/region (A) and by month (B);** 2012 data are not presented, since questionnaires were conducted in September-October 2012.

Almost half (46%) of the farmers reported at least one major (ASF-compatible) pig disease outbreak, mostly in 2008, and particularly between May and September (89.0%) (Fig 3). Locations are shown in Fig 1. There was a high interregional variability, with 92.6% of farmers in SZS reporting outbreaks, compared to just 10.0% in SK. In affected villages, 76.1% of households got affected, varying from 28.3% in SK to 86.1% in SJ (72.4% in Kakheti and 77.7% in SZS). Over half (58.0%) of the animals in the affected farms got sick or died, varying from 2.9% in SK to 72.2% in SJ (49.6% in Kakheti and 60.9% in SZS).

#### Market chain

Half of the farmers bought pigs the previous year (55.6%), usually one transaction (84.5%). Only 0.7% did four transactions or more. On average, 3.2±7.0 animals were included in each transaction, mostly piglets for fattening (52.6%). Most animals were bought on live animal markets (49.7%) and very few from commercial farms (2.8%). One quarter (25.9%) of transactions took place in March (Fig 2B). Farmers tend to collect the pigs at its origin (92.2%), rather than having them delivered.

Similarly, when selling animals, 63.7% of farmers sold pigs over the last 12 months, usually one transaction (74.8%). Only 1.6% of the famers did 4 transactions or more. On average, 6.1±3.9 animals were moved per transaction, although 34.7% sold just 1–2 pigs. Animals, mostly piglets (74.3%), were mainly sold at live animal markets (39.2%). Pigs are sold throughout the year, with peaks in December (15.5%) and May (14.3%) (Fig 2B). Most farmers (74.8%) sell at their place, rather than delivering to the buyers’ premises.

#### Home-slaughtering

**Fig 4:**
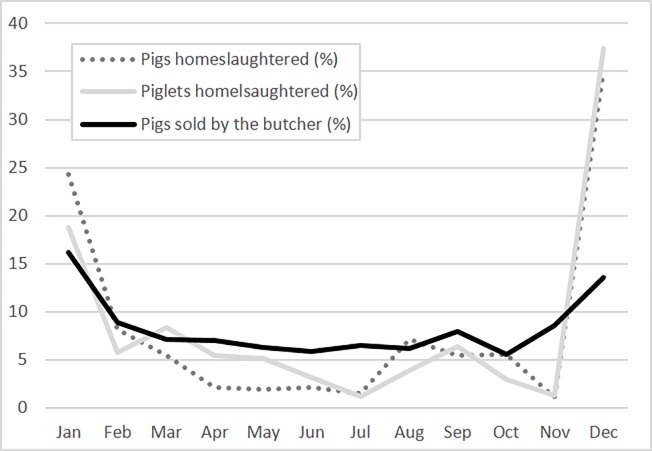
Seasonality of home-slaughtering and pigs sold by butchers in four regions in Georgia (%).

All but one farmer slaughter their pigs in their premises, mostly around Christmas (December and January), when 59.4% and 56.2% of pigs and piglets are slaughtered, respectively (Fig 4). Home-slaughtering is performed usually by a household member (83.0%) and at home (i.e. only two farmers reported to slaughter the live pigs elsewhere). Each farmer slaughters 2.6±3.9 pigs and 2.0±5.7 piglets on average, with higher numbers in Kakheti (i.e. 39.3% and 55.6% of all home-slaughtered pigs and piglets in the study).

Home-slaughtered pigs are mostly used for home consumption (91.7%). Still, 15.9% sell to middlemen, 26.0% to butchers and 48.3% of the farmers will distribute some pork to friends, neighbours or relatives. The buyers or recipients of pork products are located primarily in the same village (75.3%), or at least the same municipality (50.6%).

The main products from home-slaughtered pigs are fresh meat (71.0%) and preserved meat or fat (62.0%). On average, 57.7% of the carcass gets preserved (salted, smoked or dried), particularly in SZS (73.2%), getting consumed in 3.2±4.6 months. Leftovers from home-slaughtering are usually buried within the premises (37.6%), disposed in a pit (34.1%) or fed to dogs/cats (40.7%). Only four farmers (0.8%) reported to feed them back to the pigs.

#### Biosecurity

Most farmers feed their pigs with grain/maize (89.7%). As it is characteristic with low input systems, farmers often supplement the feeding of their animals with kitchen waste (47.8%; originating mostly from their own household (95.5%), food processing and agricultural by-products (41.7% and 37.6%). Kitchen waste is often not boiled (45.5%). Moreover, waste is sometimes thrown outside the premises (15.3%).

Household waste is mainly buried within (40.3%) or outside the household premises (22.6%). Disposal sites were presents in 41.1% of villages and even when present, only 42.6% were fenced. There is usually a fenced area where pigs can be enclosed (83.9%).

Another important biosecurity breach relates to the breeding practices: farmers either bring external boar into the farm (38.5%) or let the sows get crossed while scavenging (28.5%). Only 3.1% takes their boar to other farms, suggesting that this is a rather specialized activity, where few farmers provide the boar for the whole community. None of the farmers performs artificial insemination.

Quarantine is only applied in 34.1% of farms, and even when applied, in 64.2% of cases it is under 15 days long (i.e. the maximum incubation period for ASF, for example).

Manure, which is also a potential source of infection, is mainly used in fenced gardens and fields (69.8%), but also just dumped outside the farm (21.2%), or used in unfenced gardens/fields, thus accessible to free-ranging pigs and wild boar (20.4%).

In terms of potential interactions with wild boar, only 1% of farmers have seen wild boar close to their pigs, half of them in January. Only 3.7% of the farmers hunt wild boar (9.2% in SK).

#### FAMD and HCPC for farmers

A total of 11 qualitative and 6 quantitative variables were included in the FAMD (Table 2A). FAMD for farmers was performed taking into account the first five dimensions, which accounted for more than 50% of the variance of the data set (53.6% cumulative variance). The three variables that most contributed to the creation of the first five dimensions were: for dimension 1, “Region = Samegrelo Zemo Svaneti” (12.2%), “enclosed = no” (10.3%), “Q14 = Yes” (7.9%); for dimension 2: “Region = SJ” (12.33%), “Q14d = Yes” (9.4%), “Region = SZS” (9.2%); for dimension 3: “Region = Kakheti” (21.9%), “Region = SK” (17.3%),“Rep_Cull = Yes” (7.6%); for dimension 4: “Region = SJ” (13.7%), “Region = Kakheti” (8.7%),“Q14 = Yes” (7.6%); and for dimension 5: “Inedible_pit = Yes” (6.74%), “Rep_CullAll = Yes” (3.7%), “ASF = Yes” (3.6%).

**Table 2:** Variables (names and meanings) included in two FAMDs computed from data collected on questionnaires implemented to pig farmers and butchers in four regions of Georgia, in 2012.

**A. Variables included in the FAMD from the farmers’ questionnaire**		
**Variable name**	**Variable meaning**	**QL or QT**
Region	Region where the farm is located	QL
Enclosed	Enclosed housing system used during the whole year (yes/no)	QL
Inedible_dogs	Feeding the inedible parts of the pigs to the dogs (yes/no)	QL
Inedible_pit	Disposing the inedible parts of the pigs into a pit in the village (yes/no)	QL
ASFyn	Having suffered suspected or confirmed ASF outbreaks in the past (yes/no)	QL
vAny	Vaccinating pigs against any disease (yes/no)	QL
External_boar	Allowing sows to mate with boars from other farms while scavenging outside the farmer’s premises (yes/no)	QL
Qua7	Quarantine length of more than 7 days was done last time new pigs were introduced in the farm (yes/no)	QL
Qua14	Quarantine length of more than 14 days was done last time new pigs were introduced in the farm (yes/no)	QL
Report_CullSick	Believing that if they report a suspected ASF, sick pigs will be culled (yes/no)	QL
Report_CullAll	Believing that if they report a suspected ASF, all pigs in the village will be culled (yes/no)	QL
Income	Percentage of household income originated from pig rearing	QT
Consumer_House	Percentage of home-slaughtered pig products destined for home consumption	QT
Consumer_Friends	Percentage of home-slaughtered pig products offered to friends, relatives or neighbours	QT
Npigs	Number of pigs in the farm	QT
Altitude	Altitude of the farm	QT
Grain	Percentage of feed coming from grain or maize	QT
**B. Variables included in the FAMD from the butchers’ questionnaire**		
processed	Buying processed pig products or not (yes/no)	QL
p_syst	Predominant (>50%) system of purchased pigs. It could be: bakyard farms (bf), live animal markets (lam), middle men (mm), or sparse (mix, <50% in all categories)	QL
sf_orig	Predominant (>50%) size of the farm of origin. It could be: 0 sows (no_sows), 1–5 sows (small_org), >5 sows (medium_org), or <50% in all categories (sparse_org)	QL
dist_orig	Location of the farm of origin, categorised according to the predominant (more than 50%) origin. Categories: same village (s_vig), same municipality (s_mun), another municipality or region (a_munreg), sparse_dist (mix, <50% in all categories)	QL
sell_RH	Selling their pork to restaurants and hotels (yes/no)	QL
custom_orig	Origin of the customers, categorised according to the predominant (>50%) origin, i.e. same village (cs_vig), same municipality (cs_mun), another municipality or region (ca_munreg), or <50% in all categories (sparse_custom)	QL
livepiglets.b	Buying live piglets (yes/no)	QL
localB	Buying 100% local breeds (yes/no)	QL
SLinButcher	Whether they slaughter the pigs in their butcher premises or not	QL
livepigs	Number of live pigs bought	QT
livepiglets	Number of live piglets bought	QT
daysBFRsl	Days the pigs are kept alive before slaughter	QT
shareP	Share of pork in their overall business compared to other meats in terms of quantity (%)	QT
enclosed	Percentage of pigs bought from a production system where pigs were enclosed all year around	QT

QL: qualitative; QT: quantitative

**Fig 5:**
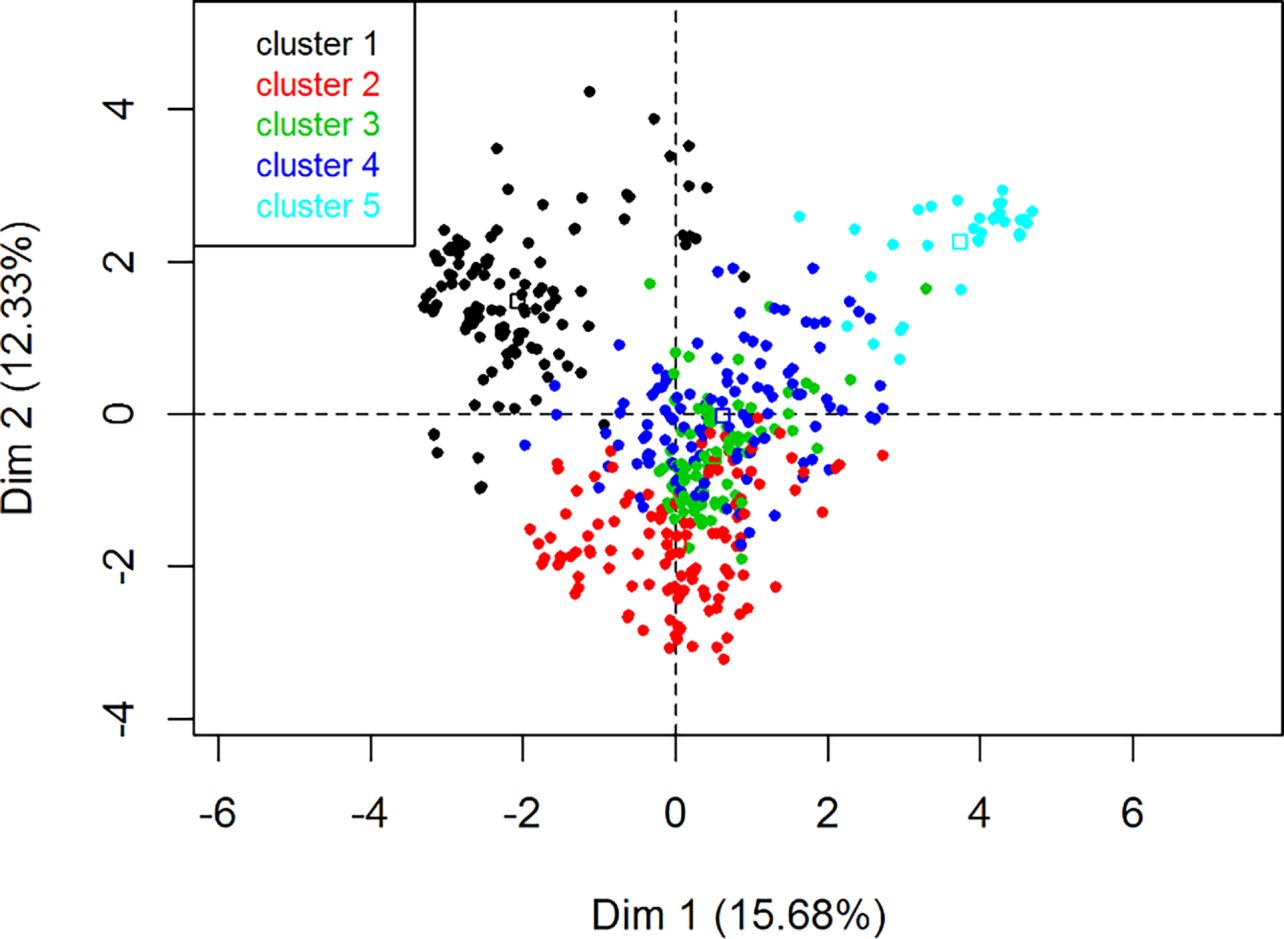
Map of individuals (farmers) and related clusters chosen during a hierarchical clustering on principal components based on the gain in within-inertia. Each round dot represents a farmer. Clusters were formed according to the similarities in answers in the collected variables of the questionnaire; thus, the closer the dots are, the more similar the answers of those farmers were. Each square dot represents the centroid of each specific cluster.

**Fig 6:**
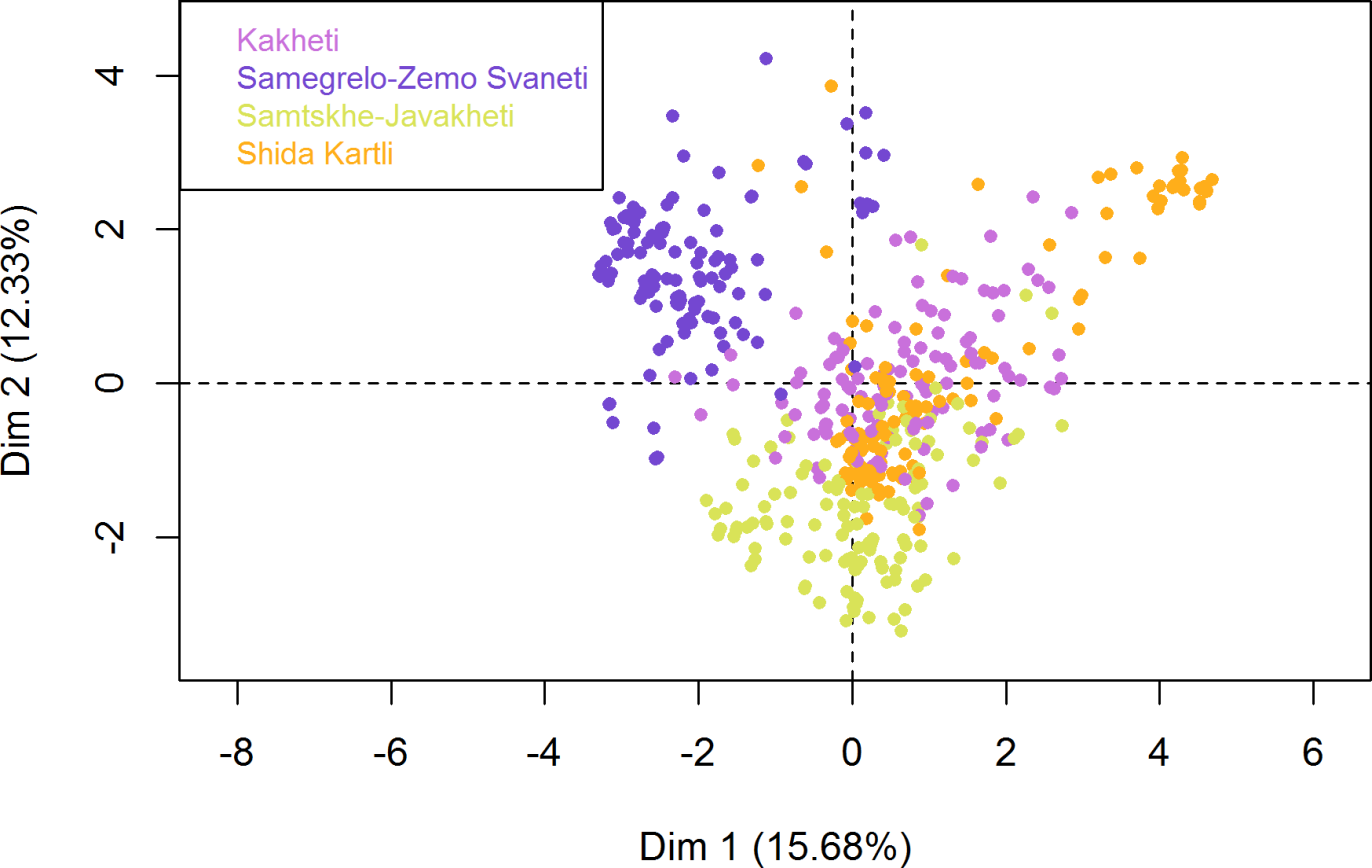
Contribution of the variable Region to the first two dimensions of the farmers’ FAMD. Dimension one explains 15.68% of the variability, whereas dimension two explains 12.33%. Each round dot represents a butcher. The closer the dots are, the more similar the answers of those farmers were. This figure indicates that butchers living within the same region (coloured) had similar answers to the questionnaire.

**Fig 7:**
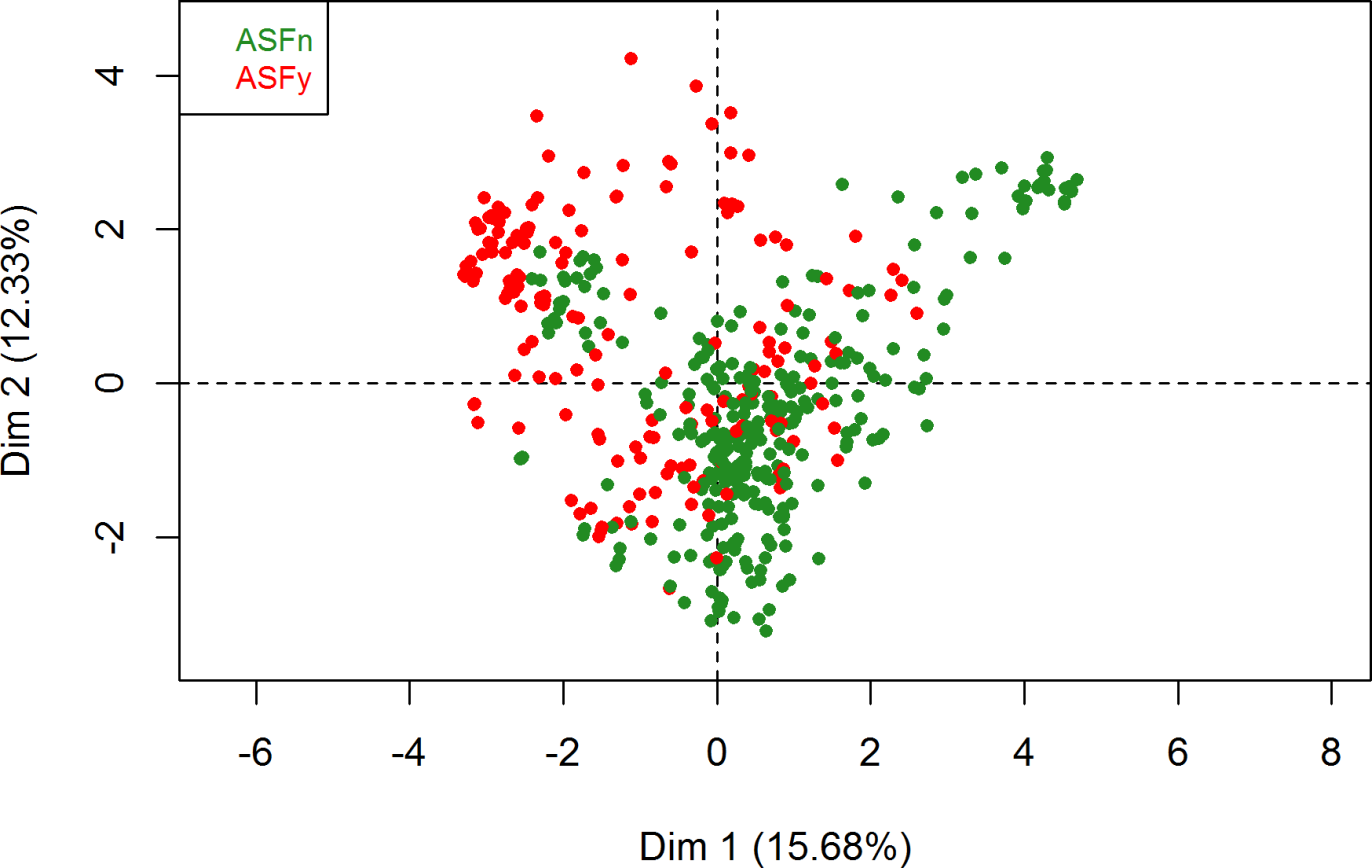
Distribution of ASF occurrence in the individual plot of the farmers’ FAMD. Each round dot represents a farmer. The closer the dots are, the more similar the answers of those farmers were. This figure indicates that farmers that reported African swine fever outbreaks had similar answers to the questionnaire, as they are represented in the figure as being closer together.

Regarding the HCPC, the partitioning of the farmers’ tree clustering resulted in a selection of five clusters (Fig 5). The rest of the dimensions were ignored, as the noise increase associated with including them in the FAMD did not justify the scarce added variation explanation they could provide (the fewer noise in the data, the more stable the HCPC clustering becomes). The main qualitative variable by which clusters were split in the first four clusters was region, indicating that main differences in our population are associated with region (Fig 6). Fig 7 shows the distribution of ASF occurrence in the individual plot of the FAMD. When compared with Fig 6, we can visually observe the overlapping of ASF occurrence and the different regions, i.e., “ASF = Yes” mainly overlaps with SZS region. A detailed description of the qualitative variables that contributed to the creation of the clusters is presented in Table 3. For example, if we look at cluster 1 and the variable region we observe that 99.2% of the interviewees living in the region SZS belong to cluster 1 (% of the sample in the cluster), 95.9% of the interviewees included in cluster 1 belong to the SZS region (% of category in cluster), and 24.2% of the interviewees of the study pertain to SZS (% of category in sample).

**Table 3:** Description of the most influencing qualitative variables in the selection of clusters of a hierarchical cluster analysis.

Qualitative variable	Variable outcome	% of the sample in the cluster ^(^^1^^)^	% of category in cluster ^(^^2^^)^	% of category in sample ^(^^3^^)^
**Region**	SZS	99.2	95.9	24.2
**Enclosed**	No	68.4	88.5	32.4
**ASF**	Yes	52.9	75.4	35.7
**Report_CullSick**	No	33.7	95.9	71.3
**Vaccinate**	No	45.9	59	32.2
**CLUSTER 2**				
**Region**	SJ	97.6	100	25.7
**Inedible_pit**	No	31.9	84.4	66.3
**Vaccinate**	Yes	29.7	80.3	67.8
**Q14**	No	27	95.1	88
**CLUSTER 3**				
**Region**	SK	72.6	100	25.5
**ASF**	No	27.5	95.6	64.3
**Enclosed**	Yes	26.5	96.7	67.6
**Report_CullSick**	No	25.1	96.7	71.3
**Vaccinate**	Yes	25.8	94.4	67.8
**CLUSTER 4**				
**Region**	Kakheti	97.5	99.2	24.6
**External boar**	No	38.4	70.3	44.4
**Enclosed**	Yes	32.2	89.8	67.6
**Report_CullSick**	Yes	42.14	53.2	28.8
**CLUSTER 5**				
**Q14**	Yes	58.6	97.1	11.9
**Report_Cull All**	Yes	34.8	91.4	18.9
**Q7**	Yes	29.6	97.1	23.6
**Region**	SK	25	88.6	25.5
**Inedible pit**	Yes	18.9	88.6	33.7

^1.^ Percentage of the sample in the cluster = % of individuals with the variable outcome in the study population who are in the cluster.

^2.^ Percentage of category in cluster = % of individuals in the cluster with the variable outcome.

^3.^ Percentage of category in sample = % of the variable in the study population.

All quantitative variables included in the FAMD were statistically significantly linked to the variable cluster. The largest effect sizes (measured by the proportion of total variation explained by that variable effect, eta square) were found in the variables Altitude and Consumer_Home (0.5 and 0.19, respectively). The quantitative variables that had a higher v.test were, for cluster one: altitude (p-value <0.01, v.test = -8.83), Consumer_home (p-value<0.01, v.test = 3.1) and Consumer_Friends (p-value<0.01, v.test = 3.1); for cluster two: altitude (p-value <0.01, v.test = 14.7), income (p-value<0.01, v.test = -4.5), Consumer_home (p-value<0.01, v.test = 3.9) and grain (p-value<0.01, v.test = -3.5); for cluster three: income (p-value<0.01, v.test = 6) and grain (p-value<0.01, v.test = -4.5), for cluster four: grain (p-value<0.01, v.test = 6.7), Npigs (p-value<0.01, v.test = 5.9), altitude (<0.01, v.test = -5.7) and Consumer Friends (p-value<0.01, v.test = -3.2) and for cluster five: Consumer_Home (p-value<0.01, v.test = -9), Consumer_Friends (p-value<0.01, v.test = -3.6) and grain (p-value = 0.03, v.test = 2.2).

### Butchers

Detailed results are presented in S2 Table. Within their butchering business, almost half of their income (47%) comes from selling pork. They sell meat from mostly local breeds (90.5%). Fattened pigs and sows represent 94.3% of the sold animals (the rest being piglets). Most butchers do not sell piglets (81.7%), which is only common in SK, where they buy more per year than the other three regions combined.

Over one fourth of butchers report to buy already processed products made from local pigs to sell them at their shop, mainly fresh sausages and minced meat, particularly in SK. It is less common for butchers to process the meat themselves, with some regional exceptions. None of the butchers sold wild boar meat.

Butchers home-slaughter the animals either at the farm of origin (65.5%) or at their own place (69.6%), with only 13.8% reporting use of slaughterhouses. About one forth slaughters the pigs immediately on arrival to their premises, while the rest will keep them alive for an average of 2.5±1.8 days. Leftovers from butchering are usually buried or disposed in a pit.

The pigs are mostly bought directly from backyard farms that have 0–5 sows, both form scavenging and enclosed systems, but also through middlemen or live animal markets. The latter is particularly common in SK, and rare in SJ. Pigs usually originate from nearby farms (within the same or adjacent municipalities). It is not uncommon for the butchers to own pigs (7%).

Customers tend to be mainly individuals, but 42.1% also sell to restaurants, particularly in SJ. In SK, butchers often sell to food processing industries (56.7%). Customers originate mainly within the same town or village or in another location within the same municipality (50.4%). Selling to customers from Tbilisi is particularly common in SK.

There is also a marked seasonality that matches home-slaughtering patterns, with the months around Christmas being the most popular months to sell pork (January 92.0% and December 75.2%).

#### FAMD and HCPC for butchers

A total of 9 qualitative and 5 quantitative variables were included in the FAMD (out of 130 available variables), as listed in Table 2B. In both FAMDs, quantitative variables were standardized to balance the influence of each variable for the study prior to conduct the FAMD analysis. FAMD for butchers was performed taking into account the first three dimensions, which accounted for almost 50% of the variance of the data set (49.5% cumulative variance). The three variables that most contributed to the creation of the first three dimensions were: for dimension 1, “Region = SZS” (8.5%), (7%), “livepiglets.b = yes” (7%), “custom_orig = sparse_custom”; for dimension 2: “Region = SJ” (9.5%), “livepiglets.b = yes” (8.7%), “Region = SZS” (6.3%); for dimension 3: “Region = Kakheti” (17%), “sell_RH = yes” (11.4%), “Region = SJ” (8.9%).

**Fig 8:**
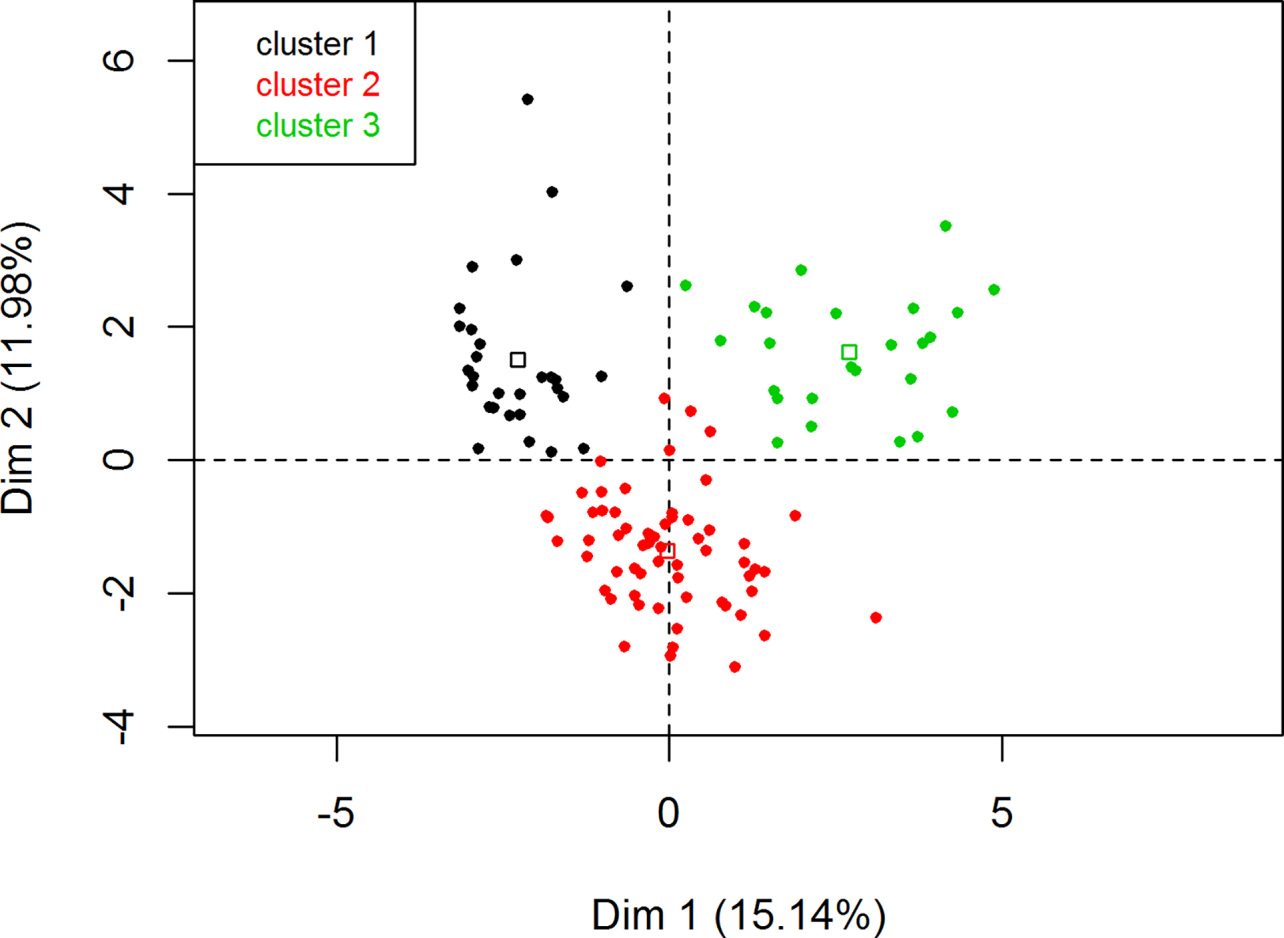
Map of individuals (butchers) and related clusters chosen during a hierarchical clustering on principal components based on the gain in within-inertia. Each round dot represents a butcher. Clusters were formed according to the similarities in answers in the collected variables of the questionnaire; thus, the closer the dots are, the more similar the answers of those butchers were. Each square dot represents the centroid of each specific cluster.

Regarding the HCPC, the partitioning of the butchers’ tree clustering resulted in a selection of three clusters (Fig 8). The rest of the dimensions were ignored. A detailed description of the qualitative variables that influenced the most in characterizing the creation of the clusters and that obtained a positive v.test can be found in Table 4.

**Table 4:** Description of the most influencing qualitative variables in the selection of clusters of a hierarchical cluster analysis.

Variable	Variable outcome	% of the sample in the cluster ^(^^1^^)^	% of category in cluster ^(^^2^^)^	% of category in sample ^(^^3^^)^
CLUSTER 1				
Region	SZS	93.5	100	26.7
Size farm origin	Small (1–5)	49.1	93.1	47.4
Local breed	Yes	38.8	89.7	57.8
Origin customer	Another municipality/region	100	17.24	4.31
CLUSTER 2				
Region	SJ	100	10.3	21.5
Buy live piglets	No	64.2	98.4	81.9
Region	Kakheti	90	43.5	25.9
Origin customer	Same village	69.1	75.8	58.6
CLUSTER 3				
Buy live piglets	Yes	85.7	72	18.1
Region	SK	70	84	25.9
Origin customer	Sparse	59.3	64	23.2
Local breed	No	42.9	84	42.2
Size farm origin	Medium (>5)	72.7	32	9.5

^1.^ Percentage of the sample in the cluster = % of individuals with the variable outcome in the study population who are in the cluster;

^2.^ Percentage of category in cluster = % of individuals in the cluster with the variable outcome;

^3.^ Percentage of category in sample = % of the variable in the study population.

All quantitative variables included in the FAMD were statistically significantly linked to the variable cluster. The larger effect sizes were found in the variables enclosed and live piglets (0.6 and 0.54, respectively). The most statistically significantly different quantitative variables (p value<0.05) that described each of the clusters were, for **cluster one**: enclosed (p-value <0.01, v.test = -8.2) and number of life pigs they bought (p-value<0.01, v.test = -4.4); for **cluster two**: days before slaughter (p-value <0.01, v.test = -4.6), number of life piglets they bought (p-value<0.01, v.test = -4.6), and enclosed (p-value <0.01, v.test = 4.6); for **cluster three**: number of life piglets they bought (p-value<0.01, v.test = 7.8) and enclosed (p-value <0.01, v.test = 3.2).

The clusters identified imply three distinct groups of butchers: 1) Cluster 1 in SZS, where butchers tend to buy from small farms with local breeds located and with customers coming from other municipalities/regions; 2) Cluster 2 in SJ and Kakheti, where butchers do not buy no live piglets and customers tend to originate within the same village; and 3) Cluster 3 linked to SK, where butchers buy more often both live piglets and commercial breeds from medium-size farms, and tend to have customers both local and from other regions.

### Awareness

Detailed information on is presented in the S3 Table. Both farmers and butchers learned about ASF mostly through TV (69.8% and 78.5%, respectively) or a veterinarian (63.2% and 75.9%).

In terms of their knowledge about ASF transmission, 68.1% of farmers and 74.1% of butchers knew that infected animals will transmit the disease. Still, the wind (40.1% and 29.3% for farmers and butchers, respectively), the water (30.9% and 25.0%), mosquitoes (16.9% and 13.8%), and bad vaccines (5.6% and 6.9%) were wrongly blamed by many as a source of infection or a mean of transmission. Also, the non-zoonotic nature of ASF was unknown to 12.6% of the interviewed farmers and 7.8% of the butchers. This seems to indicate that butchers are better informed about the ASF infection pathways than the farmers.

In terms of what will happen following the reporting of an outbreak, there seems to be very diverse opinions on what policy will be applied. While most farmers and butchers believe nothing will happen (60.5% and 60.3%, respectively), still, many think that some sort of stamping out policy will be applied. Only in SZS, where most outbreaks were reported by farmers, there seems to be a consensus that no action will be taken (91.0% of farmers and 87.1% of butchers).

## Discussion

In this study, two questionnaires were developed to characterize the Georgia swine industry in terms of husbandry, management and marketing systems, paying particular attention to those factors that could play a role in disease transmission, particularly ASF. When trying to identify ways to manage the spread of infectious diseases, it becomes critical to thoroughly understand and quantify the practices by farmers and others along the value chain. The results obtained allow to identify and quantify some of the most important gaps, e.g. in biosecurity, that would need to be addressed for a better prevention of disease spread. While biosecurity and management practices have already been described for industrial pig farming [12–15], few studies have focused on traditional production systems, and, except for Relun *et al*. [16] in Corsica, most of them covered non-European countries [17–20]. Moreover, no study has looked into the practices by butchers. This study provides detailed insights into the management of pig farming in the Caucasus, including quantified data on practices, which could help to better understand and prevent the potential spread of infectious diseases.

### Farmers

Our results show that pig farming in Georgia is not a professional and specialized sector. It is also not a homogeneous sector and farming practices and profiles vary considerably from region to region. Indeed, HCA results confirmed that the different production systems/strategies used by Georgian farmers to rear pigs closely matched the four regions where the study was conducted. Overall, pig farming is characterized by smallholdings of few animals, with low inputs (e.g. kitchen waste is a common practice), outdated technologies (e.g. none of the households use artificial insemination and only 1.9% hire personnel), and poor biosecurity, which all translates into low outputs and productivity (e.g. just 7.9 piglets per sow and year). In fact, pig farming is the major economic activity only for 9.3% of farmers. It is a highly traditional and seasonal activity, with mostly local breeds (86.2%), predominant scavenging systems in some regions, and traditional home-slaughtering for domestic consumption, mostly around Christmas. Both pigs and pork follow informal value chains, with very few pig transactions (usually just one per year) of few animals, mostly piglets, and mostly through live animal markets, while the products from home-slaughtered pigs are often distributed among neighbours, friends and relatives, mostly as fresh meat, but also salted or smoke dried.

In terms of farm biosecurity, many frequent breaches have been identified that can contribute to disease spread, i.e. scavenging systems, swill feeding, lack of disposal sites, the dumping of manure and household waste outside the premises, moving breeding boar from household to household and the lack of quarantines being applied.

The use of scavenging systems presents probably the biggest challenges, particularly in SZS, where only 12.3% of pigs are enclosed all year round. In such systems, the limited contact between farmers and their pigs may increase the time it takes to detect diseases. In addition, it favours the spread of diseases through direct contact while mixing with other herds or wild boar [16, 21–23], or by scavenging on infected food, carcasses or other fomites [24]. Moreover, confining pigs has been shown to lead to lower prevalence of diseases like zoonoses and internal parasites [25]. However, these systems are difficult to change, since they go deep into the traditions and roots of the communities and allow the production of pigs with minimum investments (in terms of feeding, fencing or personnel). Moreover, the meat produced that way is highly appreciated by consumers.

Meat and meat products are often the result of home-slaughtering, with no veterinary supervision, which constitutes a risk of spreading diseases, particularly zoonoses [26–28]. Moreover, a number of swine diseases can be transmitted through the consumption of infected pork and by-products, i.e. ASF, foot and mouth disease, classical swine fever and porcine reproductive and respiratory syndrome virus [29–30]. Swill feeding with kitchen waste that has not been heat-treated is relatively widespread in Georgia, despite being identified as a major risk factor for pig diseases [31–34]. In other cases, home-slaughtering and household waste are sometimes just dumped or disposed in unfenced waste disposal sites or landfills without any prior treatment, which has been identified as a risk factor when accessible to scavenging pigs or wild boar [35–36]. Similarly, the farmland usage or dumping of slurry can also contribute to the spread of infectious diseases by contaminating water, soil and food [37–39].

Pigs and pork reportedly moved mostly through informal routes, i.e. given and sold to friends, relatives and neighbours, often involving middlemen, and sometimes over long distances, which could translate into rapid spread of diseases. Still, most farmers only bring few new animals (3.2 on average) once a year. The profile of the pig trade dynamics resulting from the questionnaires have been analysed and discussed in detail by Kukielka et al. [40].

Despite the above biosecurity breaches, animal health management was generally good, with frequent consultations with veterinarians (8.1 per year), which translates in the majority of farmers taking the right measures when they encounter sick or dead pigs, thus avoiding further disease transmission. The exception would be the use of vaccines, which could be considerably improved. Vaccination coverage is highly variable throughout the country (42%-92.5% depending on the region), showing a direct relationship with the mortality observed, i.e. the highest the vaccination coverage, the lowest the mortality.

Wild boar can play a very important role in the transmission of pig diseases [41]. However, unlike in other similar studies [23], their connection to domestic pigs seems to be uncommon in Georgia, i.e. few sightings of wild boar close to pig farms and few hunting farmers. Even the connection via wild boar meat sold by butchers was not proven.

When analysing the reported occurrence of outbreaks consistent with ASF, one can observe a marked seasonality towards summer months when pig density increases. This is consistent with observations in other ASF-affected countries, e.g. the Russian Federation [42]. The majority of the outbreaks were reported in SZS, which is easily appreciated when comparing Figs 6 and 7, and similar to WAHIS official outbreak data, which show the same region reporting 42.3% of the outbreaks in the four selected regions (data not shown). This may be explained by the fact that SZS has predominantly scavenging systems that allow the disease to move easily from one household to the next. Inversely, SK presents both the lowest number of outbreaks and the highest percentage of enclosed husbandry systems. Finally, the peak of outbreaks in 2008 and its gradual decline thereafter is consistent with the time when ASF first spread throughout Georgia (2007–2008). It also implies that the disease likely remained present in the country unreported.

#### Farmers by region

The HCPC analysis confirmed that there are five major production and husbandry strategies, which match the four regions where the study was conducted (i.e. Cluster 1: SZS; Cluster 2: SJ; Clusters 3 and 5: SK; and Cluster 4: K).

Kakheti farmers keep the highest mean number of animals (16.9). Indeed, out of 13 farms interviewed with over 50 animals, 9 are placed in Kakheti. Animals are mostly enclosed all year round (89.2%). Kakheti farmers use commercial breeds (23.1%), hired personnel (4.2%), have their own breeding boar (42.3%), and work as butchers (7.5%) more often than the other three regions. Although these characteristics suggest a more commercial farming structure, paradoxically, the household income from pig rising is low (19.6%) and the use of commercial feed is the lowest (22.5%). Health management is also deficient, the vaccination rate is low, with 44.4% of farmers not vaccinating against any pig disease. Although only 26.7% of the households use kitchen waste, the waste is not boiled in 71.4% of the farms. Kakheti home-slaughters twice the amount of pigs and piglets than the other regions. Farmers do not share much meat with relatives, friends, etc., but mostly sell the meat to butchers (29.4%). It is unique to Kakheti that farmers never process the meat into sausages or boiled/heat-treated products (unlike the other regions). Farmers often use grain or maize as one of the ingredients in the feed (97.5%).

Samegrelo-Zemo Svaneti’s pig sector is characterized by scavenging systems (87.7%) throughout the year using local breeds (94.8%) and by having the smallest mean size litter (6.8 piglets per litter). As expected, sows get pregnant while scavenging (56.8%) and animals disappear more often than in other regions. Most farmers (58.0%) never vaccinate against any disease, which may influence the high mortality rates (2.4±4.1 pigs per farm). This is consistent with the fact that SZS is the region with most outbreaks reported (Fig 1). The low vaccination rate may be related to the fact that animals scavenge most of the time, which will make it difficult to gather them for vaccination. SZS’s farmers use live animal markets as the primary place both for buying and selling animals, but unlike in other regions, they hardly ever sell to butchers (0.4% compared to 19.6% for Georgia). Also unlike other regions, they buy and sell replacement sows very often (37.9% and 7.7%, respectively). In SZS, it is more common to home-slaughter piglets than pigs. It is the only region where it is more common to make sausages or dry/salt/smoke the meat than eating fresh pork. SZS is the region that uses kitchen waste to feed pigs more frequently.

Samtskhe-Javakheti has the lowest household income from pig farming (15.9%), and stands in between Kakheti and SZS in terms of the use of local breeds and the popularity of scavenging systems, which is allowed from April to October by 28.8% of farmers. Farms are located at a higher altitude than the average altitude of sampled farms. In terms of health management, they have a high vaccination rate (80.8%). In SJ, they home-slaughtered less animals than in the other regions (2.4±1.9 compared to an average of 4.6±9.6 in all regions). Perhaps because of that it mostly goes for home-consumption and relatives, friends, etc. within the village and almost nothing to restaurants (9% vs. 3.1% nationally) or butchers (0.8% vs. 26.0%). The feeding of pigs uses a wide range of sources, with grain/maize, kitchen waste, food processing by-products and agricultural by-products all registering over 50% usage. The region has the lowest implementation and length of quarantines (21.9% and 3.1±6.7 days, respectively).

Shida Kartli has the most professional farming, as demonstrated by the highest household income from pig farming (31.7%). Despite having the lowest number of animals per farm (8.7±9.2), this is compensated by having the most prolific sows (9.8 piglets per sow). They use mostly local breeds (93.3%) in permanently enclosed systems (98.3%). Health management seems best among all four regions, with a high vaccination rate (92.5%), which translates in the lowest mortality (1.7%). The farmers in SK are also the ones interacting most frequently with the local veterinarians (in general and in the specific case of pigs getting sick or dying). In spite of this, SK’s farmers report the highest rates for wrongdoing following the finding of a sick or dead pig, e.g. slaughter for home consumption (8.7%) or to sell the meat of dead pigs (10%). SK’s farmers use live animal markets as the primary place for buying animals, but unlike other regions, they also buy often from commercial farms (22.5% compared to 5.5% at country level) and they sell mostly to other smallholders (43.8% compared to 18.7% at country level). Another peculiarity of the region is that farmers never purchase pigs fattened half way (8.7% in the rest of the studied regions). SK’s home-slaughtering seems more business-oriented than in other regions, with fewer farmers reporting self-consumption and the highest rate of selling to butchers (63.3% compared to the national average of 26.0%). In the same line, the consumers of the home-slaughtered animals are more likely to be in other municipalities or regions. Finally, less piglets are home-slaughtered than other regions (0.5±2.5 vs. 2±5.7). When feeding pigs, SK’s farmers use more commercial feed (50.8%) and food processing by-products, and fewer kitchen waste (22.5%, which is heat-treated in 66.6% of the households). The sources of kitchen waste include not just the own household, but also market, neighbours and restaurant leftovers being often used, unlike other regions. SK is the region using external boar for mating purposes more often (68.8%), as well as the region where quarantines are most often used (43.3%), which are as well the longest (8.2 days±12.2). The percentage of farmers hunting wild boar is higher (9.2%).

### Butchers

Butchers, as opposed to pig farmers, have butchering as their major occupation and source of income. As expected, they follow similar patterns to the farmers in terms of breeds (mostly local), seasonality (more sells around Christmas), source farms (small farms with 0–5 sows), and trade patterns (rather local).

Butchers can also play an important role in the disease dynamics, particularly for those diseases that are transmitted via infected meat, including zoonoses. Butchers could either sell infected pork that could eventually reach pigs and other susceptible species via swill feeding or dumped kitchen waste, or through mismanagement of the leftovers from butchering (e.g. thrown away or fed back to pigs). In our study, only three butchers (1.7%) reported pigs getting sick while awaiting slaughter; nevertheless, the risk of selling meat of animals that were asymptomatic or during the incubation period is patent. In any case, the role of butchers in long distance spreading of potentially infected meat does not seem to be too important, given that only less than 3% of butchers sell to customers from other (non-adjacent) municipalities or regions.

It would be very informative to implement similar type of questionnaires to other secondary stakeholders along the pig and pork value chain, who also haven important role in disease transmission, such as middlemen, live animal market managers, etc. [43–46]. Hunters were considered for inclusion in this study due to their role connecting domestic pigs and wildlife (particularly wild boar), but it was decided against because of the low wild boar density, and hunting not being widespread among the farmer community (as demonstrated by this study).

### Recommendations

Our results highlight the need for improved biosecurity, husbandry practices and production systems. Existing systems should gradually shift to a pig sector that is more commercialized, professional and vertically integrated through better educated farmers. The promotion of farmers’ associations could help by allowing better access to information and trainings on biosecurity and health management, good husbandry practices, access to markets, feed, soft loans or microcredits, veterinary and financial services, and to lobby government for support.

Specifically on disease management, training stakeholders on disease transmission, biosecurity, and the importance of timely reporting is key. This training should go beyond farmers and butchers, reaching also middlemen and other actors along the value chain, as well as field veterinarians. Our results also highlight a breach in communication of official control policies to farmers. Thus, consequences of reporting disease outbreaks should be clarified and better explained to farmers. Moreover, reporting rates could be increased if incentives were to be offered as a way to encourage farmers’ cooperation and to help them deal with the hardship associated to disease outbreaks. Moreover, sustainable outbreak control strategies adapted to the Georgian reality must be developed in consultation with pig owners and other stakeholders, moving away from traditional approaches, which may not be possible in countries without the necessary resources. For instance, in the absence of compensation funds, the traditional stamping out may not be possible. Instead, a modified stamping out strategy, quarantine and controlled marketing of animal may be more effective.

There is a need to understand pig and pork value chains throughout the country to identify and mitigate risks throughout the chain, as recommended by FAO [47]. Farm and animal identification and censuses for pigs are also essential.

### Limitations

Selecting interviewees according to some pre-specified rules may have introduced selection bias in the study. The farmer-butcher ratio of 80:20 was based on expert’s opinion, since such statistics are not available in the country. Other criteria, such as preferably targeting farms with larger number of pigs, aimed to ensure the inclusion in the study of those who may play a more important role. Targeting interviewees who live in towns of certain characteristics (e.g. mountainous areas) was done to prevent such populations being excluded.

Farmers or butchers whose practices are irregular or illegal may have refused to take part in the survey or their answers may reflect what they feel is expected, instead of giving a truthful answer. Still, a surprisingly large number of irregular practices were reported. There is an interesting finding when comparing the results in Kakheti with those of the pilot study conducted by official veterinarians also in Kakheti (Beltrán-Alcrudo, unpublished). There appears to be a better compliance to animal health management and biosecurity related questions when official veterinarians conduct the interviews, e.g. higher vaccination rates for all diseases (i.e. CSF (79.2 vs 40.2; Erysipela: 79.2 vs. 25.6; Aujezsky: 50.0 vs 17.1; pasteurellosis: 50.0 vs 18.0), more frequent consultations with veterinarians when encountering sick pigs (82.8 vs. 67.3%), more frequent boiling of swill feed (87.5 vs. 28.6%), more frequent and longer quarantine (83.3 vs. 39.5%, and 15.4 vs. 7.1 days) and even higher ASF awareness through all official means, and only lower for unofficial means (i.e. rumours/neighbours: 20.0 vs. 39.5). This seems to indicate that the official status of veterinarians, pushes farmers to answer towards what is expected. This is why we believe it is important to have questionnaires conducted by private non-official veterinarians, who are known and trusted.

Although the response rate was high, one cannot neglect the fact that 30 out of 41 and 22 out of 24 questions had a response rate under 95% for the farmers and butchers, respectively. This could lead to answers non representative of the whole study area population. As questionnaires covered a period of 12 months, recall bias could also be influencing our results.

The questionnaires were conducted by 27 veterinarians. Although efforts were made to ensure the clarity of the questions and interviewers were trained, the interviewers may have unintentionally influenced the responses, so interviewer bias cannot be completely excluded. Moreover, the meaning of some questions may have been altered when translating the materials. Questionnaires were closed, as opposed to open questions that could allow interviewees to provide more details answers or clarifications.

Only four administrative units were selected for the study (out of a total of 9 regions, 1 city, and 2 autonomous republics). Moreover, villages, farms and butchers were not drawn randomly, so the practices described in this paper cannot be assumed to be representative of the whole Georgian pig producers and butchers, but rather an overview of the husbandry and management practices in the country. Finally, the inclusion of farms was based on the voluntary participation of farmers; and thus, selection bias was probably introduced.

## Conclusion

In countries like Georgia, rearing pigs is a very common and traditional practice in rural areas, representing an important source of meat and often generating valuable cash income. Despite most pigs being kept in backyards and in small farms, these sectors usually do not receive much institutional support to deal with animal diseases. Moreover, disease prevention and control is most challenging in these settings due to the lower levels of disease awareness among the rural communities, low biosecurity, poor compliance to livestock related regulations (reporting, movement control, certifications, vaccination, etc.) and lack of animal identification and traceability. However, not all Georgian pig herds are exposed to the same level of risk.

Because of the above and the fact that veterinary services often lack personnel, equipment and funds to control the disease, there is a need to engage all stakeholders (pig keepers, butchers, middlemen, wild boar hunters and private veterinarians) and empower them to deal with pig diseases. Such approach needs to be based on a thorough understanding of people’s behaviours, trade patterns and management practices involved in smallholder pig production and processing, which are largely unknown and highly diverse within the country. To gather such information in a quantifiable manner, questionnaires were developed for pig keepers and butchers covering biosecurity and husbandry practices, market chains, wild boar interactions, awareness status and socio-economic aspects.

The present study of the management of extensive pig production in Georgia increases our knowledge on traditional management practices. The analysis of the questionnaires will allow quantifying biosecurity gaps and risky behaviours, develop risk profiles, and identify critical control points across the market chain where to implement mitigation measures. Ultimately this will allow the design of realistic and sustainable prevention, surveillance and control strategies, largely based on awareness campaigns and trainings and realistic biosecurity principles.

## Supporting information

S1 Database Databases used for the factorial analyses of mixed data (FAMD) of farmers questionnaires. (CSV)

S2 Database Databases used for the factorial analyses of mixed data (FAMD) of butchers questionnaires. (XLS)

S1 Table Results of pig farmers’ surveys in four regions of Georgia. (DOCX)

S2 Table Results of the butchers’ surveys in four regions of Georgia. (DOCX)

S3 Table Results of the awareness questions for both pig farmers and butchers in four regions of Georgia. (DOCX)

S1 Appendix Questionnaire forms for pig keepers and butchers in English and Georgian. (XLSX)
