# Supplementary material for: Descriptive and multivariate analysis of the pig sector in Georgia and its implications for disease transmission
Source: PLoS One. 2018 Aug 24;13(8):e0202800. doi: 10.1371/journal.pone.0202800 (PMC6108502; doi:10.1371/journal.pone.0202800)
Supplement: S1 Table — (DOCX) [file pone.0202800.s003.docx]

**S1 Table. Results of pig farmers’ surveys in four regions of Georgia**

Herd composition, socioeconomic aspects and production systems

| **Variable** | **Kakheti** | **Samegrelo Zemo-Svaneti** | **Samtskhe Javakheti** | **Shida Kartli** | **Overall total** |
| --- | --- | --- | --- | --- | --- |
| **Age-gender composition of the herd** | | | | | |
| Number of sows | **2.3** | 2.2 | 1.2 | 1.3 | 1.7 |
| Number of boar | **1.2** | 0.9 | 0.6 | 0.2 | 0.7 |
| Number of fattened pigs | **3.9** | 1.7 | 2.4 | 3.3 | 2.8 |
| Number of piglets eaten or sold | **9.6** | 4.2 | 5.5 | 3.9 | 5.8 |
| Total number of animals | **16.9** | 9.1 | 9.6 | 8.7 | 11.0 |
| **Income from pig raising (%)** | 19.6 | 26.5 | 15.9 | **31.7** | 23.4 |
| **Also work as butcher** | **7.5** | 1.6 | 0.8 | 5.0 | 3.7 |
| **Who takes care of the pigs** | | | | | |
| Wife | 54.2 | 62.3 | 80.8 | 80.8 | 69.6 |
| Husband | 79.2 | 54.9 | 76.0 | 93.3 | 75.8 |
| Kids | 7.5 | 16.4 | 47.2 | 40.0 | 27.9 |
| Other family members | 17.5 | 33.6 | 26.4 | 19.2 | 24.2 |
| Hired personnel | **4.2** | 1.6 | 0.8 | 0.8 | **1.9** |
| **Breed of pigs (whether commercial or local)** | | | | | |
| Local breed | 76.9 | **94.8** | 80.2 | 93.3 | 86.2 |
| Commercial breed | **23.1** | 5.2 | 19.8 | 6.8 | 13.8 |
| **Production system** | | | | | |
| Pigs enclosed all year round | 89.2 | 12.3 | 71.2 | **98.3** | 67.6 |
| Pigs allowed to scavenge during the day, but return every night | 10.8 | **82.8** | 27.2 | 0.0 | 30.6 |
| Pigs scavenge for days or months | 0.0 | **4.9** | 1.6 | 1.7 | 2.3 |
| **Piglets per sow** | 7.5 | 6.8 | 7.7 | **9.8** | 7.9 |
| **Average village altitude (m)** | 569.4 | 612.5 | **1632.5** | 1003.7 |  |

Health management by region

| **Variable** | **Kakheti** | **Samegrelo Zemo-Svaneti** | **Samtskhe Javakheti** | **Shida Kartli** | **Overall total** |
| --- | --- | --- | --- | --- | --- |
| **Mortality (%)** | 1.9 | **2.6** | 0.6 | 0.1 | 1.3 |
| **Vaccination against…** | | | | | |
| None | 44.4 | **58.0** | 19.2 | **7.5** | 32.0 |
| Classical swine fever | 40.2 | 36.1 | 70.4 | **83.3** | 57.8 |
| Erysipela | 25.6 | 32.8 | 60.0 | **75.8** | 48.9 |
| Pasteurellosis | 18.0 | 2.5 | 15.2 | **41.7** | 19.3 |
| Aujeszky | 17.1 | 3.4 | 5.6 | **28.3** | 13.5 |
| Other (please specify) | 14.5 | 0.0 | 0.0 | 1.7 | 5.0 |
| **Consultations with veterinarian** | 8.2 | 7.6 | 5.3 | **11.3** | 8.1 |
| **Action taken by farmers the last time a pig got sick** | | | | | |
| Treat the animal/s yourself | 77.0 | 60.3 | 80.0 | **89.3** | 76.3 |
| Consult a veterinarian | 67.3 | 66.9 | **96.4** | 91.2 | 80.4 |
| Slaughter for home-consumption | 1.0 | 1.7 | 0.0 | **8.7** | 2.7 |
| Slaughter and sell the meat | 1.0 | 0.0 | 0.9 | **3.5** | 1.3 |
| Sell the animal/s alive | 0.0 | 0.0 | 0.0 | **1.0** | 0.2 |
| Culling and disposal | 3.8 | 3.3 | **6.4** | 1.9 | 3.8 |
| Nothing | 8.7 | **14.0** | 1.8 | 1.0 | 6.5 |
| **Action taken by farmers the last time a pig died** | | | | | |
| Burying | 62.5 | 69.4 | 58.1 | **80.0** | 66.3 |
| Thrown away | 0.0 | 9.4 | **12.9** | 10.0 | 7.8 |
| Disposal in a pit | **37.5** | 27.1 | 22.6 | 20.0 | 28.3 |
| Burning | 0.0 | 1.2 | 3.2 | 0.0 | 1.2 |
| Fed to the pigs | 0.0 | 0.0 | 0.0 | 0.0 | 0.0 |
| Consumption of the meat | 0.0 | 2.4 | 3.2 | 0.0 | 1.8 |
| Selling the meat | 0.0 | 0.0 | 0.0 | **10.0** | 0.6 |
| Collected by dedicated services | 0.0 | 1.2 | 0.0 | 0.0 | 0.6 |
| Contact your private veterinarian | 5.0 | 3.5 | 22.6 | **50.0** | 10.2 |
| Contact veterinary authorities | 5.0 | 2.4 | **9.7** | 0.0 | 4.2 |

The pig market chain

| **Variable** | **Kakheti** | **Samegrelo Zemo-Svaneti** | **Samtskhe Javakheti** | **Shida Kartli** | **Total** |
| --- | --- | --- | --- | --- | --- |
| **Type of provider/buyer** | | | | | |
| Piglets | 64.0 / 68.7 | 44.4 / 85.3 | 46.2 / 80.3 | 60.9 / 67.4 | **53.6 / 74.3** |
| Replacement sows | 15.5 / 4.1 | 37.9 / 7.7 | 18.4 / 3.8 | 21.8 / 2.4 | 23.9 / 4.3 |
| Ready to slaughter pigs | N.A. / 24.5 | N.A. / 6.9 | N.A. / 13.0 | N.A. / 27.0 | N.A. / 19.1 |
| Boar | 8.4 / 2.6 | 10.6 / 0.0 | 22.4 / 2.9 | 17.3 / 3.2 | 13.8 / 2.3 |
| Pigs fattened half way | 12.1 / N.A. | 7.2 / N.A. | 13.0 / N.A. | 0.0 / N.A. | 8.7 / N.A. |
| Total | 29.9 / 26.6 | 29.5 / 20.6 | 22.5 / 22.3 | 18.0 / 30.5 |  |
| **Type of pig purchased / sold** | | | | | |
| Live animal market | 54.4 / 43.1 | 55.6 / 50.5 | 48.0 / 48.3 | 34.8 / 21.6 | **49.7 / 39.2** |
| Middleman | 19.3 / 27.0 | 26.6 / 45.2 | 16.6 / 8.6 | 23.0 / 11.2 | 21.6 / 21.8 |
| Smallholder | 23.0 / 3.5 | 17.1 / 3.6 | 32.7 / 16.8 | 19.7 / 43.8 | 22.9 / 18.7 |
| Butcher | N.A. / 25.3 | N.A. / 0.4 | N.A. / 25.9 | N.A. / 23.1 | N.A. / 19.6 |
| Commercial | 3.4 / 0.0 | 0.7 / 0.0 | 2.7 / 0.0 | 22.5 / 0.3 | 5.8 / 0.1 |
| Food Manufacturer | N.A. / 0.0 | N.A. / 0.0 | N.A. / 0.4 | N.A. / 0.0 | N.A. / 0.1 |
| Total | 29.8 / 26.7 | 29.5 / 20.6 | 22.5 / 22.3 | 17.9 / 30.4 |  |

Home-slaughtering practices by region

| **Variable** | **Kakheti** | **Samegrelo Zemo-Svaneti** | **Samtskhe Javakheti** | **Shida Kartli** | **Overall total** |
| --- | --- | --- | --- | --- | --- |
| Pigs home-slaughtered per farm | 4.1 | 1.7 | 1.6 | 3.0 | 2.6 |
| Piglets home-slaughtered per farm | 4.6 | 2.3 | 0.8 | 0.5 | 2.0 |
| **Type of consumers of the home-slaughtered products** | | | | | |
| Home consumption | 93.3 | 95.9 | 99.2 | **78.3** | 91.7 |
| Relatives, friends, neighbours | **24.4** | 61.5 | 52.0 | 55.0 | 48.3 |
| Butcher/shop | **29.4** | 11.5 | 0.8 | 63.3 | 26.0 |
| Middleman | 16.8 | **11.5** | 16.3 | 19.2 | 15.9 |
| Restaurant/bar | 2.5 | **5.7** | 0.0 | 4.2 | 3.1 |
| **Location of consumers of the home-slaughtered products** | | | | | |
| Same village | 59.1 | 74.0 | 88.2 | 77.1 | 75.3 |
| Same municipality | 38.7 | 34.0 | 48.2 | 76.3 | 50.6 |
| Adjacent municipality | 9.7 | 6.0 | 8.2 | 19.5 | 11.2 |
| Non-adjacent municipality | 1.1 | 1.0 | 1.8 | 8.5 | 3.3 |
| Another region | 3.2 | 4.0 | 4.5 | 5.1 | 4.3 |
| Tbilisi | 19.4 | 18.0 | 19.1 | 19.5 | 19.0 |
| I do not know | 8.6 | 5.0 | 2.7 | 4.2 | 5.0 |
| **Type of products from home-slaughtered pigs** | | | | | |
| Fresh meat | 77.4 | 48.3 | 77.1 | 83.7 | 71.0 |
| Dried/smoked/salted meat or fat | 60.9 | 73.7 | 58.1 | 53.8 | 62.0 |
| Fresh fat | 15.7 | 14.4 | 37.1 | 34.6 | 24.9 |
| Sausage | 0.0 | 51.4 | 10.5 | 26.9 | 19.9 |
| Boiled/heat treated meat or fat | 0.0 | 29.7 | 25.7 | 20.2 | 18.8 |
| **Inedible parts** | | | | | |
| Fed to dogs / cats | 52.2 | 17.2 | 40.3 | 54.2 | 40.7 |
| Buried within premises | 9.6 | 52.5 | 43.5 | 43.3 | 37.6 |
| Disposal in a pit | 24.3 | 40.2 | 16.1 | 55.8 | 34.1 |
| Buried outside premises | 9.6 | 4.9 | 8.9 | 11.7 | 8.7 |
| Dispose as household waste | 9.6 | 0.0 | 8.1 | 5.8 | 5.8 |
| Thrown away outside premises | 2.6 | 0.0 | 7.3 | 5.0 | 3.7 |
| Collected by companies, municipality, etc. | 0.0 | 0.0 | 2.4 | 4.2 | 1.7 |
| Burned inside premises | 0.9 | 0.8 | 1.6 | 1.7 | 1.2 |
| Fed back to pigs | 0.0 | 0.8 | 0.0 | 2.5 | 0.8 |
| Burned outside premises | 0.0 | 0.0 | 0.0 | 0.8 | 0.2 |
| Other (please specify) | 0.0 | 0.0 | 0.0 | 0.8 | 0.2 |
| Nothing left after slaughtering | 11.3 | 0.0 | 3.2 | 0.0 | 3.5 |

Biosecurity practices

| **What do you feed your pigs** | **Kakheti** | **Samegrelo Zemo-Svaneti** | **Samtskhe Javakheti** | **Shida Kartli** | **Overall total** |
| --- | --- | --- | --- | --- | --- |
| Grain/maize | 97.5 | 88.5 | 93.4 | 82.5 | 89.7 |
| Kitchen waste | 26.7 | 72.1 | 68.8 | 22.5 | 47.8 |
| Food processing by-product | 17.5 | 31.2 | 53.6 | 64.2 | 41.7 |
| Commercial feed | **22.5** | 48.4 | 36.8 | **50.8** | 39.6 |
| Agricultural byproducts | 35.0 | 26.2 | 56.8 | 31.7 | 37.6 |
| Butcher leftovers | 0.0 | 3.3 | 1.6 | 3.3 | 2.0 |
| **Origin of kitchen waste/food scraps** | | | | | |
| Own household | 97.8 | 96.7 | 92.3 | 95.9 | 95.5 |
| Market | 0.0 | 18.2 | 12.0 | 29.9 | 17.1 |
| Other households / neighbours | 2.2 | 4.1 | 7.7 | 33.0 | 9.7 |
| Restaurant | 0.0 | 0.8 | 1.7 | 4.1 | 1.8 |
| Other. Please specify | 0.0 | 2.5* | 1.7 | 7.2 | 3.2 |
| **Do not boil leftovers** | 71.4 | 43.4 | 46.6 | 33.7 | 45.5 |
| **Household waste disposal** | | | | | |
| Buried within premises | 20.0 | 61.5 | 30.0 | 48.7 | 40.3 |
| Buried outside premises | 27.8 | 2.5 | 12.5 | 48.7 | 22.6 |
| Collected by municipality | 39.1 | 9.0 | 22.5 | 8.4 | 19.5 |
| Burned | 6.1 | 11.5 | 26.7 | 32.8 | 19.3 |
| Thrown outside premises | 11.3 | 18.0 | 26.7 | 5.0 | 15.3 |
| Thrown within premises | 1.7 | 1.6 | 10.8 | 6.7 | 5.2 |
| **Waste disposal site in the village** | | | | | |
| No disposal site available | 29.7 | 77.0 | 68.9 | 59.2 | 58.9 |
| Fenced disposal site | 21.2 | 12.3 | 1.6 | 40.0 | 18.7 |
| Unfenced disposal site | 16.1 | 11.5 | 26.2 | 4.2 | 14.5 |
| Buried at disposal site | 22.9 | 1.6 | 0.8 | 27.5 | 13.1 |
| Burned at disposal site | 13.6 | 0.8 | 4.9 | 8.3 | 6.8 |
| **Availability of a fenced place** | 89.2 | 70.5 | 78.7 | 97.5 | 83.9 |
| **External boar to cross** | | | | | |
| Yes | 22.5 | 34.7 | 32.3 | 68.8 | 38.5 |
| No, sows get crossed while scavenging | 10.8 | 56.8 | 26.3 | 16.1 | 28.5 |
| No, I own a boar | 42.3 | 18.6 | 18.2 | 11.8 | 23.3 |
| No, I own a boar and take it to other premises for breeding | 5.4 | 1.7 | 1.0 | 4.3 | 3.1 |
| No, I use artificial insemination | 0.0 | 0.0 | 0.0 | 0.0 | 0.0 |
| No, I have no breeding animals (sows or boar) | 23.4 | 0.8 | 26.3 | 2.2 | 13.1 |
| **Quarantine the last time the farmer purchased pigs** | | | | | |
| No quarantine | 60.5 | 68.9 | 78.1 | 56.7 | 65.9 |
| *Mean* | 7.1 | 7.2 | 3.1 | 8.2 | 6.4 |
| **Use of manure** | | | | | |
| Use it in fenced garden/fields | 59.2 | 69.7 | 76.0 | 74.2 | 69.8 |
| Dump it outside my premises | 20.8 | 21.3 | 10.4 | 31.7 | 21.2 |
| Use it in unfenced garden/fields | 16.7 | 12.3 | 36.0 | 17.5 | 20.4 |
| Store it, e.g. in a pit | 1.7 | 4.9 | 5.6 | 8.3 | 5.1 |
| Sell it/give it to others. | 1.7 | 0.0 | 0.8 | 2.5 | 1.2 |

*1 farmer reported that his food scraps come from a military base
