# Supplementary material for: Descriptive and multivariate analysis of the pig sector in Georgia and its implications for disease transmission
Source: PLoS One. 2018 Aug 24;13(8):e0202800. doi: 10.1371/journal.pone.0202800 (PMC6108502; doi:10.1371/journal.pone.0202800)
Supplement: S2 Table — (DOCX) [file pone.0202800.s004.docx]

**S2 Table. Results of the butchers’ surveys in four regions of Georgia**

|  | **Kakheti** | **Samegrelo Zemo-Svaneti** | **Samtskhe Javakheti** | **Shida Kartli** | **Overall tot**a**l** |
| --- | --- | --- | --- | --- | --- |
| **Share of pork in business (%)** | 61.6 | 28.0 | 52.3 | 47.2 | 47.1 |
| **Number of animals bought** | | | | | |
| Live pigs | 75.9 | 25.3 | 103.7 | 65.9 | 65.8 |
| Live piglets | 2.9 | 1.8 | 0.0 | 10.6 | 4.0 |
| **Breed of the animals bought** | | | | | |
| Local breeds | 88.7 | 96.8 | 92.4 | 84.3 | 90.5 |
| Commercial breeds | 11.3 | 3.2 | 7.6 | 15.7 | 9.5 |
| **If you buy life pigs, where do you slaughter them?** | | | | | |
| Home-slaughter at farm of origin | 63.3 | 45.2 | 80.0 | 76.7 | 65.5 |
| Home-slaughter at my place | 53.3 | 80.7 | 68.0 | 73.3 | 69.9 |
| Slaughterhouse | 10.0 | 16.1 | 12.0 | 16.7 | 13.8 |
| **Process into products / Buy?** | | | | | |
| Fresh sausage/kubati | 0.0 / 17.9 | 19.4 / 32.3 | 4.0 / 4.0 | 6.7 / 53.3 | 7.9 / 28.1 |
| Minced meat | 3.6 / 7.1 | 3.2 / 0.0 | 40.0 / 16.0 | 16.7 / 63.3 | 14.9 / 21.9 |
| Dried/smoked/salted meat/fat | 3.6 / 3.6 | 12.9 / 16.1 | 4.0 / 4.0 | 10.0 / 6.7 | 7.9 / 7.9 |
| **Days alive before slaughtering** | | | | | |
| Not applicable (slaughter at the farm of origin or buy carcasses) | 40.0 | 9.7 | 20.0 | 26.7 | 24.1 |
| Immediately (0 days) | 50.0 | 22.6 | 24.0 | 16.7 | 28.5 |
| More than one day | 10 | 67.7 | 56 | 56.7 | 47.4 |
| Mean | 0.7 | 2.3 | 0.8 | 2.0 | 1.5 |
| **Leftovers from butchering** | | | | | |
| Cleaning company | 10.3 | 22.6 | 30.8 | 26.7 | 21.4 |
| Disposed in a pit | 41.4 | 51.6 | 69.2 | 50.0 | 50.5 |
| Thrown away | 13.8 | 3.2 | 15.4 | 6.7 | 8.7 |
| Burned (at the market) | 0.0 | 0.0 | 7.0 | 10.0 | 3.9 |
| Buried | 37.9 | 51.6 | 30.8 | 73.3 | 51.5 |
| Rendered | 17.2 | 3.2 | 0.0 | 0.0 | 5.8 |
| Fed back to pigs | 3.4 | 0.0 | 0.0 | 0.0 | 1.0 |
| Sold | 0.0 | 0.0 | 0.0 | 0.0 | 0.0 |
| Other | 6.9 | 0.0 | 0.0 | 0.0 | 1.9 |
| **Type of pig providers** | | | | | |
| Middleman | 20.7 | 38.7 | 52.0 | 76.7 | 47.0 |
| Directly from farm or backyard | 89.7 | 80.7 | 96.0 | 86.7 | 87.8 |
| Live animal market | 37.9 | 19.4 | 8.0 | 63.3 | 33.0 |
| Slaughterhouse | 3.4 | 6.5 | 0.0 | 13.3 | 6.1 |
| Own pigs | 6.9 | 0.0 | 0.0 | 20.0 | 7.0 |
| Hunters (wild boar) | 0.0 | 0.0 | 0.0 | 0.0 | 0.0 |
| **Size of pig herd of origin** | | | | | |
| Farms without sows | 53.8 | 22.6 | 72.0 | 70.0 | 56.6 |
| Backyard (1-2 sows) | 73.1 | 58.1 | 92.0 | 66.7 | 71.4 |
| Small farm (3-5 sows) | 26.9 | 54.8 | 80.0 | 63.3 | 56.3 |
| Medium farm (6-10 sows) | 0.0 | 6.5 | 40.0 | 36.7 | 20.5 |
| Large farm (>10 sows) | 0.0 | 0.0 | 24.0 | 23.3 | 11.6 |
| **Production system at the farm of origin** | | | | | |
| Pigs enclosed all year round | 100.0 | 13.9 | 88.7 | 95.0 | 72.1 |
| Pigs scavenge during the day | 0.0 | 80.6 | 9.9 | 3.0 | 25.5 |
| Pigs scavenge several days/months | 0.0 | 5.5 | 1.4 | 2.0 | 2.4 |
| **Origin of pigs /Origin of customers** | | | | | |
| Same village/town | 89.3 / 93.1 | 87.1 / 51.6 | 80.0 / 92.0 | 100.0 / 93.3 | 89.5 / 81.7 |
| Other village/town within same municipality | 35.7 / 24.1 | 35.5 / 41.9 | 84.0 / 80.0 | 56.7 / 60.0 | 51.8 / 50.4 |
| Adjacent municipality | 17.9 / 13.8 | 12.9 / 19.4 | 28.0 / 12.0 | 36.7 / 26.7 | 23.7 / 18.2 |
| Another municipality | 10.7 / 0.0 | 6.5 / 0.0 | 0.0 / 8.0 | 20.0 / 0.0 | 9.6 / 1.7 |
| Another region | 0.0 / 0.0 | 0.0 / 9.7 | 0.0 / 0.0 | 10.0 / 0.0 | 2.6 / 2.6 |
| Tbilisi (only customers) | - / 10.3 | - / 6.5 | - / 4.0 | - / 26.7 | - / 12.2 |
| Unknown | 0.0 / 0.0 | 0.0 / 0.0 | 0.0 / 40.0 | 0.0 / 0.0 | 0.0 / 8.7 |
| **Type of costumers** | | | | | |
| Individual costumers | 100.0 | 95.8 | 80.0 | 100.0 | 98.2 |
| Restaurant and hotels | 13.8 | 25.8 | 87.5 | 50.0 | 42.1 |
| Food processing | 0.0 | 9.7 | 0.0 | 56.7 | 17.5 |
| Other | 0.0 | 3.2 | 0.0 | 0.0 | 0.9 |
