# Supplementary material for: Descriptive and multivariate analysis of the pig sector in Georgia and its implications for disease transmission
Source: PLoS One. 2018 Aug 24;13(8):e0202800. doi: 10.1371/journal.pone.0202800 (PMC6108502; doi:10.1371/journal.pone.0202800)
Supplement: S3 Table — (DOCX) [file pone.0202800.s005.docx]

**S3 Table. Results of the awareness questions for both pig farmers and butchers in four regions of Georgia**

| **Variable for pig farmers / butchers** | **Kakheti** | **Samegrelo Zemo-Svaneti** | **Samtskhe Javakheti** | **Shida Kartli** | **Overall total** |
| --- | --- | --- | --- | --- | --- |
| **How farmers learnt about African swine fever** | | | | | |
| Radio | 6.7 / 6.7 | 13.9 / 16.1 | 17.6 / 20.0 | 3.3 / 3.3 | 10.5 / 11.2 |
| TV | 72.3 / 80.0 | 59.8 / 61.3 | 75.2 / 88.0 | 71.7 / 86.7 | 69.8 / 78.5 |
| Newspaper | 6.7 / 3.3 | 26.2 / 25.8 | 23.2 / 16.0 | 19.2 / 13.3 | 18.9 / 14.7 |
| Veterinarian | 39.5 / 30.0 | 64.8 / 87.1 | 62.4 / 92.0 | 86.7 / 96.7 | 63.2 / 75.9 |
| Local authorities | 16.0 / 26.7 | 17.2 / 25.8 | 6.4 / 44.0 | 5.8 / 3.3 | 11.3 / 24.1 |
| Rumors / Neighbours | 39.5 / 33.3 | 32.0 / 29.0 | 11.2 / 0.0 | 14.2 / 3.3 | 24.0 / 17.2 |
| Leaflets / Posters | 2.5 / 0.0 | 10.7 / 12.9 | 12.8 / 36.0 | 4.2 / 3.3 | 7.67 / 12.1 |
| Internet | 2.5 / 0.0 | 4.1 / 0.0 | 0.0 / 8.0 | 2.5 / 3.3 | 2.3 / 2.6 |
| Church | 0.0 / 0.0 | 0.0 / 0.0 | 0.0 / 0.0 | 0.8 / 0.0 | 0.21 / 0.0 |
| Other | 0.0 / 3.3 | 0.0 / 0.0 | 4.0 / 0.0 | 0.8 / 0.0 | 1.23 / 0.9 |
| **ASF transmission** | | | | | |
| Bad vaccine | 3.4 / 10.0 | 6.6 / 6.5 | 1.6 / 0.0 | 10.8 / 10.0 | 5.6 / 6.9 |
| Through the wind | 39.5 / 43.3 | 33.6 / 35.5 | 41.6 / 8.0 | 45.8 / 26.7 | 40.1 / 29.3 |
| Bringing infected animal | 70.6 / 63.3 | 61.5 / 71.0 | 69.6 / 88.0 | 70.8 / 76.7 | 68.1 / 74.1 |
| Borrowing infected equipment | 13.4 / 20.0 | 18.0 / 25.8 | 27.2 / 36.0 | 25.8 / 23.3 | 21.2 / 25.9 |
| Feeding infected pork/products to pigs | 21.0 / 36.7 | 36.9 / 25.8 | 42.4 / 72.0 | 25.0 / 36.7 | 31.5 / 41.4 |
| Mosquitoes | 14.3 / 16.7 | 5.7 / 3.2 | 20.8 / 8.0 | 26.7 / 26.7 | 16.9 / 13.8 |
| In the water | 26.1 / 16.7 | 27.1 / 19.4 | 21.6 / 16.0 | 49.2 / 46.7 | 30.9 / 25.0 |
| Unknown | 17.6 / 13.3 | 14.8 / 9.7 | 9.6 / 4.0 | 17.5 / 13.3 | 14.8 / 10.3 |
| **If outbreak reported** | | | | | |
| Nothing | 78.8 / 46.7 | 91.0 / 87.1 | 44.3 / 25.0 | 73.1 / 76.7 | 60.5 / 60.9 |
| Culling of all pigs in the village | 27.1 / 23.3 | 4.1 / 16.1 | 17.2 / 58.3 | 28.6 / 23.3 | 19.1 / 28.7 |
| Culling of only sick pigs in the village | 50.8 / 40.0 | 4.9 / 3.2 | 37.7 / 45.8 | 23.5 / 40.0 | 29.1 / 31.3 |
| Fine | 1.7 / 10.0 | 0.0 / 0.0 | 0.8 / 8.3 | 0.0 / 0.0 | 0.6 / 4.3 |
| Compensation for culled pigs | 9.3 / 3.3 | 0.0 / 0.0 | 3.3 / 0.0 | 1.7 / 0.0 | 3.5 / 0.9 |
| **Believe ASF is zoonotic** | 5.9 / 13.3 | 4.1 / 0.0 | 19.2 / 4.0 | 20.8 / 13.3 | 12.6 / 7.8 |
